# Pathological lymphangiogenesis is modulated by galectin-8-dependent crosstalk between podoplanin and integrin-associated VEGFR-3

**DOI:** 10.1038/ncomms11302

**Published:** 2016-04-12

**Authors:** Wei-Sheng Chen, Zhiyi Cao, Satoshi Sugaya, Maria J. Lopez, Victor G. Sendra, Nora Laver, Hakon Leffler, Ulf J. Nilsson, Jianxin Fu, Jianhua Song, Lijun Xia, Pedram Hamrah, Noorjahan Panjwani

**Affiliations:** 1 Program in Cell, Molecular and Developmental Biology, Sackler School of Graduate Biomedical Sciences, Tufts University, Boston, Massachusetts 02111, USA; 2 New England Eye Center/Department of Ophthalmology, Tufts University, Boston, Massachusetts 02111, USA; 3 Department of Laboratory Medicine, Section of Microbiology Immunology and Glycobiology, Sölvegatan, Lund University, Lund SE-22184, Sweden; 4 Centre for Analysis and Synthesis, Department of Chemistry, Lund University, Lund SE-22100, Sweden; 5 Cardiovascular Biology Research Program, Oklahoma Medical Research Foundation, Oklahoma City, Oklahoma 73104, USA; 6 Department of Biochemistry and Molecular Biology, University of Oklahoma Health Sciences Center, Oklahoma City, Oklahoma 73104, USA; 7 Department of Developmental, Molecular and Chemical Biology, Tufts University, Boston, Massachusetts 02111, USA

## Abstract

Lymphangiogenesis plays a pivotal role in diverse pathological conditions. Here, we demonstrate that a carbohydrate-binding protein, galectin-8, promotes pathological lymphangiogenesis. Galectin-8 is markedly upregulated in inflamed human and mouse corneas, and galectin-8 inhibitors reduce inflammatory lymphangiogenesis. In the mouse model of corneal allogeneic transplantation, galectin-8-induced lymphangiogenesis is associated with an increased rate of corneal graft rejection. Further, in the murine model of herpes simplex virus keratitis, corneal pathology and lymphangiogenesis are ameliorated in *Lgals8*^−/−^ mice. Mechanistically, VEGF-C-induced lymphangiogenesis is significantly reduced in the *Lgals8*^−/−^ and *Pdpn*^−/−^ mice; likewise, galectin-8-induced lymphangiogenesis is reduced in *Pdpn*^−/−^ mice. Interestingly, knockdown of VEGFR-3 does not affect galectin-8-mediated lymphatic endothelial cell (LEC) sprouting. Instead, inhibiting integrins α1β1 and α5β1 curtails both galectin-8- and VEGF-C-mediated LEC sprouting. Together, this study uncovers a unique molecular mechanism of lymphangiogenesis in which galectin-8-dependent crosstalk among VEGF-C, podoplanin and integrin pathways plays a key role.

## Abstract (web summary)

Pathological lymphangiogenesis is associated with various eye diseases. Here the authors show that a carbohydrate-binding protein, galectin-8, promotes pathological lymphangiogenesis in the eye by regulating the crosstalk among VEGF-C, podoplanin and integrin pathways, and thus may represent a useful therapeutic target.

## Graphical abstract

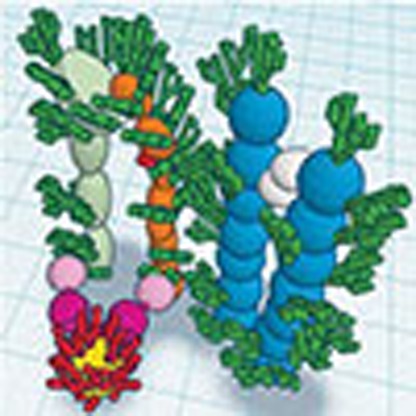

Lymphatic vessels are essential for preservation of fluid balance, nutrient absorption and immune surveillance. Lymphangiogenesis is associated with diverse pathological conditions including metastatic dissemination, solid organ graft rejection, type 2 diabetes, obesity, hypertension, lymphedema and chronic wound healing^1^. Pathological lymphangiogenesis is also associated with various diseases of the eye including corneal graft rejection, herpetic keratitis, dry eye disease, ocular allergy and glaucoma^2^,^3^,^4^,^5^,^6^,^7^. Indeed, recent studies suggest that lymphatic vessel invasion in and around primary tumours rather than invasion of blood vessels, is the key prognostic marker of the aggressiveness of various types of cancers^8^, and that the growth of lymphatic vessels is also the major reason of corneal graft rejection^3^. The key regulatory signalling axis that induces lymphangiogenesis is vascular endothelial growth factor receptor-3 (VEGFR-3) and its ligand, VEGF-C.

Recent studies have demonstrated that the members of the galectin family of mammalian lectins characterized by a carbohydrate recognition domain (CRD) with affinity for β-galactoside-containing glycans, play a critical role in hemangiogenesis. In this respect, we have shown that a member of galectin family, galectin-3, modulates VEGF-A-induced angiogenic response by binding via its CRD to the *N*-glycans of integrin αvβ3 and VEGFR-2 and subsequently activating angiogenic signalling pathways^9^,^10^. Galectin-8 is a tandem-repeat type member of the galectin family. It contains two different CRDs. The N-terminal CRD prefers α2,3-sialyl glycans and mainly contributes to its unique carbohydrate-binding specificity^11^,^12^,^13^. *In vitro* studies have shown that galectin-8 binds to podoplanin (PDPN) and that the lectin promotes adhesion and haptotaxis of lymphatic endothelial cells (LECs)^14^. However, the direct evidence that galectin-8 exerts its biological functions through PDPN is lacking. In fact, Cueni and Detmar^14^ speculated that contribution of the interaction of galectin-8 with PDPN in the modulation of LEC migration and adhesion is most likely minor. Also, based on the findings that both unglycosylated and extensively glycosylated PDPN-Fc inhibit LEC adhesion and migration *in vitro*, it has been suggested that the interactions with PDPN ligands on the surface of LECs do not depend on PDPN glycosylation^15^. To date, the biological relevance of carbohydrate-dependent galectin-8/PDPN interactions is still elusive and more direct studies involving the use of galectin-8 knockout (KO) and PDPN KO mice have not been reported.

PDPN is a unique transmembrane receptor protein^16^. It is expressed by LECs but not blood ECs and promotes blood-lymph separation. Mice lacking PDPN have leaky lymphatic vessels and congenital lymphedema^17^,^18^. *In vitro* studies have shown that PDPN expression in LECs is required for lymphatic capillary tube formation as well as VEGF-A-induced cell migration^19^,^20^. The critical role of extracellular domain of PDPN in lymphangiogenesis has been demonstrated by studies showing that PDPN-Fc and the functional blocking antibody against extracellular domain of PDPN inhibit LEC migration and tube formation *in vitro* and suppress lymphangiogenesis in inflamed mouse corneas *in vivo*^15^,^21^. The extracellular domain of PDPN is heavily glycosylated, and *O*-glycosylation as well as sialylation are critical for PDPN-mediated blood-lymph separation and platelet aggregation^18^,^22^,^23^.

Integrins are a major class of adhesion receptors. It is well-established that angiogenic signals induced by VEGF and the interplay between VEGF receptors and integrins expressed on endothelial cells play an important role in the process of angiogenesis. Integrins that are well-defined for their role in lymphangiogenesis include α1β1, α2β1, α4β1, α5β1 and α9β1 (reviewed in Chen *et al*.^24^). Activation of integrin β1 by collagen and fibronectin promotes VEGFR-3 activation^25^. Conversely, inhibition of integrin α5β1 but not αvβ3 attenuates VEGF-C-induced VEGFR-3 activation^26^. In addition to extracellular matrix proteins and growth factor receptors, integrins are glycosylated and interact with distinct members of galectin family in a glycan-dependent manner^10^,^27^. Although integrins, VEGFR-3 and PDPN are glycosylated like most cell surface receptors, more direct studies on the role of carbohydrate-dependent function of integrins, PDPN and VEGF-C/VEGFR-3 in the regulation of lymphangiogenesis have not been reported.

In the current study, using multiple approaches involving the use of galectin-8 mutants lacking carbohydrate-binding activity, KO mice, specific sugar inhibitors of galectin-8, and siRNA knockdown of key players of lymphangiogenesis, we establish a critical role of galectin-8 and carbohydrate-mediated recognition in the process of lymphangiogenesis. We demonstrate that galectin-8 expression is markedly upregulated in inflamed corneas, and that galectin-8 is a potent lymphangiogenic factor and a key mediator of VEGF-C signalling. Further, we show that PDPN is a key player in VEGF-C-induced lymphangiogenesis, that knockdown of PDPN interferes with integrin signalling cascades in LECs, and that galectin-8 is a critical mediator of crosstalk among VEGF-C, PDPN and integrin lymphangiogenic pathways. In addition, herpes simplex virus (HSV)-1 infection-induced pathological lymphangiogenesis is reduced in galectin-8 KO mice, and galectin-8-induced lymphangiogenesis is associated with an increased rate of graft rejection in a mouse model of allogeneic corneal transplantation. More importantly, we show here that inhibitors of galectin-8 decrease lymphangiogenesis in inflamed mouse corneas *in vivo*. This is significant considering that there is much interest in finding ways to inhibit the activities of pro-lymphangiogenic factors for preventing graft rejection, tumour metastasis and other inflammatory conditions.

## Results

### Galectin-8 is upregulated in inflamed human and mouse corneas

**Figure 1:**
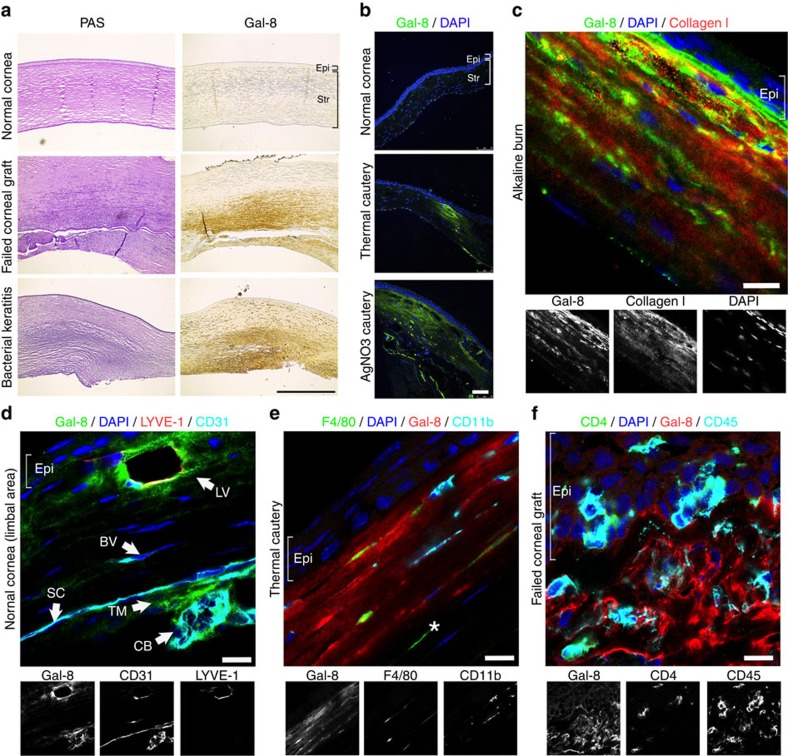
Galectin-8 is markedly upregulated in inflamed human and mouse corneas. (**a**) Normal human corneas and corneal buttons removed at keratoplasty from patients with corneal graft failure and bacterial keratitis were analysed for galectin-8 immunoreactivity in paraffin sections. Brown colour indicates positive immunostaining. PAS-stained corneas from the corresponding cases are shown in the left panel. Compared with the normal corneas, markedly greater galectin-8 immunoreactivity was detected in the corneal stroma of patients with graft failure and bacterial keratitis. (**b**) Mouse corneas subjected to silver nitrate or thermal cautery were allowed to partially heal *in vivo* and were then analysed for galectin-8 immunoreactivity in frozen sections (green). Nuclei were visualized by counterstaining with DAPI (blue). Compared with the normal corneas, markedly greater galectin-8 immunoreactivity was detected in cauterized corneas. (Immunostaining processing and colour development (**a**) and exposure time (**b**) of all images are the same). (**c**) Colocalization of galectin-8 and collagen I of corneal stromal matrix. Mouse corneas subjected to alkaline burn were allowed to heal *in vivo* for 2 weeks and were then analysed for immunoreactivity of galectin-8 (green) and type I collagen (red). Nuclei were counterstained with DAPI (blue). (**d**) Immunolocalization of galectin-8 in lymphatic vessels. Frozen sections of normal mouse corneas were analysed for immunoreactivity of galectin-8 (green), CD31 (cyan) and LYVE-1 (red). Nuclei were counterstained with DAPI (blue). (**e**–**f**) Immunolocalization of galectin-8 in infiltrating immune cells. Frozen sections of cauterized mouse corneas on postoperative day 1 (**e**) and rejected mouse corneal allografts on postoperative week 4 (**f**) were analysed for immunoreactivity of galectin-8 (red), F4/80 (green), CD4 (green), CD11b (cyan) and CD45 (cyan). Nuclei were counterstained with DAPI (blue). The white asterisk indicates a F4/80^+^ but galectin-8^−^ cell (**e**). Scale bars: 400 μm (**a**); 75 μm (**b**); and 10 μm (**c**,**d**,**e**,**f**). BV, blood vessel; CB, ciliary body; Epi, epithelium; LV, lymphatic vessel; SC, endothelium of Schlemm's canal; Str, stroma; TM, trabecular meshwork.

In corneas of patients with graft failure and bacterial keratitis, numerous inflammatory cells were detected in the stroma as highlighted by periodic acid Schiff (PAS) staining (Fig. 1a). Normal corneas expressed little galectin-8 (Fig. 1a). In contrast, robust galectin-8 immunoreactivity was detected in corneas of patients with graft failure and bacterial keratitis. Similarly, in mouse corneas treated with thermal cautery or AgNO_3_ cautery, intense galectin-8 immunoreactivity was detected in the stromal matrix, whereas in untreated control mouse corneas, galectin-8 expression was minimal (Fig. 1b). In some areas, particularly in the anterior stroma, galectin-8 and type I collagen immunoreactivity colocalized (Fig. 1c). While strong galectin-8 immunoreactivity was detected in lymphatic vessels (CD31^+^LYVE-1^+^), weak immunoreactivity of galectin-8 was observed in blood vessels (CD31^+^LYVE-1^−^, Fig. 1d). These findings are consistent with a published study showing that the mRNA and protein levels of galectin-8 are higher in LECs than in blood ECs^14^.

In inflamed mouse corneas, galectin-8 immunoreactivity was detected in macrophages (F4/80^+^CD11b^+^, Fig. 1e) and CD4^+^ T cells (CD4^+^CD45^+^, Fig. 1f). Interestingly, some F4/80^+^ cells in the posterior corneal stroma were galectin-8^-^ (Fig. 1e), suggesting that either a subset of F4/80^+^ cells express galectin-8, or the cells need to be activated to express galectin-8. While it is reasonable to suggest that cells stained positively may be the possible source of the lectin, we note that paracrine actions of galectins have been reported. In this respect, galectins secreted by one cell type may bind to the glycan receptors on the adjacent cells. Therefore, the cells that exhibit immunoreactivity with galectin-8 may not necessarily be the cells that produce the lectin. Taken together, this study demonstrates that galectin-8 is upregulated in inflamed human and mouse corneas.

### Galectin-8 promotes lymphangiogenesis *in vivo*

**Figure 2:**
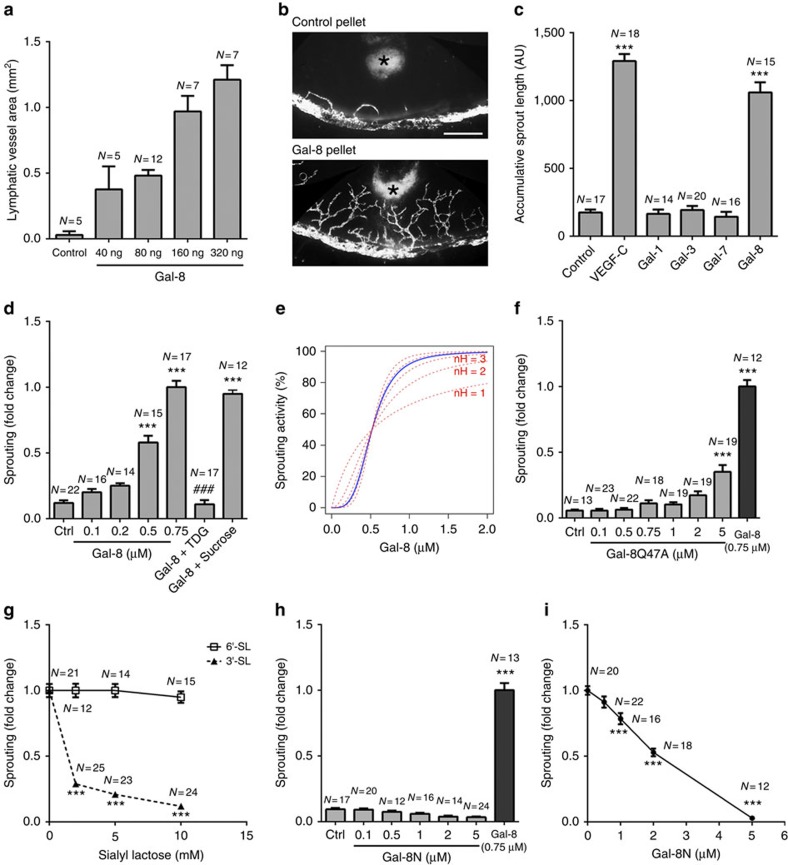
Galectin-8 promotes lymphangiogenesis *in vivo* and LEC sprouting *in vitro*. (**a**,**b**) Galectin-8 promotes lymphangiogenesis *in vivo*. Sustained-release polymer pellets containing various doses of galectin-8 were implanted in the corneas of *Prox1*-EGFP reporter mice. One week after surgery, the vessel area was calculated. Data are expressed as mean±s.e.m. (**a**). A representative fluorescence image of a cornea implanted with control or galectin-8 pellet (160 ng per pellet) is shown in **b**. Asterisks indicate pellets. Scale bar, 400 μm. (**c**–**i**) Galectin-8 promotes LEC sprouting. LEC spheroids were incubated with media containing various test agents. After 24 h, the cumulative length of the sprouts was quantified. (**c**) Galectin-8, but not galectins-1, 3 or 7, promotes LEC sprouting. Concentrations of VEGF-C and galectins are 50 ng per ml and 0.75 μM, respectively. (**d**) Galectin-8 promotes LEC sprouting in a dose- and carbohydrate-dependent manner. TDG and sucrose: 20 mM. (**e**) Galectin-8 stimulates LEC sprouting in a positively cooperative manner. Blue line indicates galectin-8-mediated LEC sprouting based on experimental results; red broken lines indicate simulated theoretical curves of different nH values (nH=1–5). (**f**) A galectin-8 mutant, Gal-8Q47A, which has lost its ability to bind to α2,3-sialylated glycans, does not promote LEC sprouting. (**g**) 3′-SL, but not 6′-SL, inhibits galectin-8-induced LEC sprouting. (**h**) Gal-8N does not promote LEC sprouting. (**i**) Gal-8N inhibits galectin-8-induced sprouting. A value of 1.0 was assigned to the sprout length of galectin-8 (0.75 μM) -treated cells. The values for all other groups are expressed as a change in the sprout length with respect to galectin-8-treated LEC spheroids. Data are plotted as mean±s.e.m. and analysed using one-way ANOVA (**c**,**d**,**f**,**h**) and Student's *t*-test (**g**,**i**). *P<0.05, ***P<0.001 versus control, ^###^*P*<0.001 versus Gal-8 (0.75 μM). ANOVA, analysis of variance; TDG, thiodigalactoside; 3′-SL, 3′-sialylated lactose; 6′-SL, 6′-sialylated lactose; Gal-8N, N-terminal CRD. The results are representative of two or more independent experiments.

The normally avascular cornea has been extensively used as the *in vivo* model to investigate the molecular mechanism of hemangiogenesis and to examine the efficacy of the inhibitors and activators of hemangiogenesis. In recent years, cornea has also proven to be an invaluable model for defining general mechanisms of lymphangiogenesis. To determine whether galectin-8 promotes lymphangiogenesis, we used the mouse corneal micropocket assay. The vessel area, representing the extent of lymphangiogenesis, was calculated 1 week after galectin-8 pellets were implanted in mouse corneas. The extent of galectin-8-mediated lymphangiogenesis increased in a dose-dependent manner, whereas control pellets had no effect (Fig. 2a,b). To further demonstrate the pro-lymphangiogenic capacity of galectin-8 *in vivo*, we employed another well-established method, the Matrigel plug assay. Matrigels containing galectin-8 or VEGF-C were injected in mice subcutaneously, where they solidified to form plugs. On day 7 post injection, the Matrigel plugs were harvested and frozen sections of the plugs were stained with anti-LYVE-1 to visualize lymphatic vessels. As expected, VEGF-C stimulated the growth of new lymphatic vessels in Matrigel, whereas PBS (control) did not (Supplementary Fig. 1a and Supplementary Methods). Similar to VEGF-C, galectin-8 also promoted robust lymphangiogenesis in Matrigel (Supplementary Fig. 1a). To determine the mitogenic effect of galectin-8 on LECs *in vivo*, corneal micropocket assays were performed using VEGF-C, galectin-8 or control pellets in *Prox1*-EGFP (enhanced green fluorescent protein) reporter mice. On day 7 post surgery, corneas implanted with various pellets were stained with anti-Ki67. In control corneas, <4% of LEC (Prox1^+^ cells) were Ki67^+^. In contrast, more than 35% of LECs were Ki67^+^ in both VEGF-C- and galectin-8-induced lymphangiogenic areas (Supplementary Fig. 1b and Supplementary Methods). These data lead us to conclude that galectin-8 is a pro-lymphangiogenic factor as assessed by two independent *in vivo* methods.

### Galectin-8 promotes LEC sprouting *in vitro*

To characterize the role of galectin-8 in the regulation of phenotypic behaviour of LECs *in vitro*, we examined the effect of galectin-8 on LEC proliferation, migration, tube formation and sprouting. In contrast to *in vivo* results, galectin-8 treatment had no effect on LEC proliferation *in vitro* (Supplementary Fig. 1c and Supplementary Methods). We reason that continuously produced galectin-8 may be required to stimulate LEC proliferation *in vitro*, as has been observed for other (lymph)angiogenic factors such as angiopoitin-1 (refs 28, 29) and sphingosine-1-phosphate^30^. Galectin-8, however, promoted cell migration and tube formation (Supplementary Fig. 1d,e and Supplementary Methods). To better characterize the molecular mechanism by which galectin-8 mediates lymphangiogenesis, we utilized an *in vitro* three-dimensional LEC sprouting assay. In the sprouting assay, galectin-8, but not galectins-1, 3 or 7, promoted LEC sprouting (Fig. 2c). The stimulatory effect of galectin-8 on LEC sprouting was concentration-dependent (Fig. 2d,e). Next, we tested whether the stimulatory effect of galectin-8 on LEC sprouting was carbohydrate-dependent. First, galectin-8-induced LEC sprouting was almost completely inhibited by thiodigalactoside (TDG), a pan inhibitor of galectins, whereas sucrose, a non-inhibiting disaccharide for galectins, had no effect (Fig. 2d). The nH (Hill coefficient) of galectin-8-induced LEC sprouting was 3.7, indicating a positively cooperative effect of galectin-8-induced LEC sprouting (Fig. 2e). Secondly, compared with wild-type (WT) galectin-8, a galectin-8 mutant, Gal-8Q47A, which has markedly reduced ability to bind α2,3-sialylated glycans^13^,^31^, required at least 10 times higher concentration to promote LEC sprouting (Fig. 2f). Thirdly, 3′-sialyllactose (3′-SL, 10 mM), which binds N-CRD but not C-CRD of galectin-8 (ref. 13), markedly inhibited galectin-8-induced LEC sprouting, whereas 6′-SL, which does not bind galectin-8, had no effect (Fig. 2g and Supplementary Fig. 2a–c). These data establish that the stimulatory effect of galectin-8 on LEC sprouting is carbohydrate-dependent and that N-CRD of galectin-8 plays a critical role in the process of galectin-8-induced lymphangiogenesis.

Next, we tested whether N-CRD can serve as a dominant negative inhibitor of galectin-8. Di/multivalent property of galectins allow them to crosslink many cell surface and extracellular matrix glycoproteins, such as integrins and growth factor receptors, to regulate signal transduction pathways^32^. Isolated CRDs, which retain their carbohydrate-binding ability but are unable to dimerize or oligomerize and crosslink cell surface receptors, may compete with the carbohydrate-binding ability of the endogenous galectins and, hence, act as a dominant negative inhibitor^10^,^33^. Published studies have shown that isolated CRDs of galectin-8 retain the carbohydrate-binding activity but manifest impaired biological activity^13^,^34^, suggesting that the biological function of the lectin is dependent on cooperative interactions of the two CRDs. As described before, N-CRD of galectin-8 (Gal-8N) is unique among galectins in exhibiting a very high affinity for α2,3-sialyl glycans^11^,^12^,^13^. To determine whether the pro-lymphangiogenic property of galectin-8 is dependent on the cooperative action of both CRDs, we tested whether N-CRD is able to promote LEC sprouting. Unlike full-length galectin-8, Gal-8N failed to induce LEC sprouting (Fig. 2h). Moreover, Gal-8N effectively inhibited galectin-8-induced LEC sprouting (Fig. 2i and Supplementary Fig. 2d–f). These results suggest that Gal-8N serves as a dominant negative inhibitor of galectin-8 and that α2,3-sialyl glycans recognized by Gal-8 N as well as cooperative action of both CRDs are required for galectin-8-induced LEC sprouting.

AKT and ERK1/2 are essential to lymphangiogenesis^35^,^36^. We, therefore, tested the activation of AKT and ERK1/2 pathways by galectin-8. Galectin-8 induced phosphorylation of AKT and ERK1/2 in a time- and dose-dependent manner (Supplementary Fig. 3).

### Galectin-8 modulates pathological lymphangiogenesis *in vivo*

**Figure 3:**
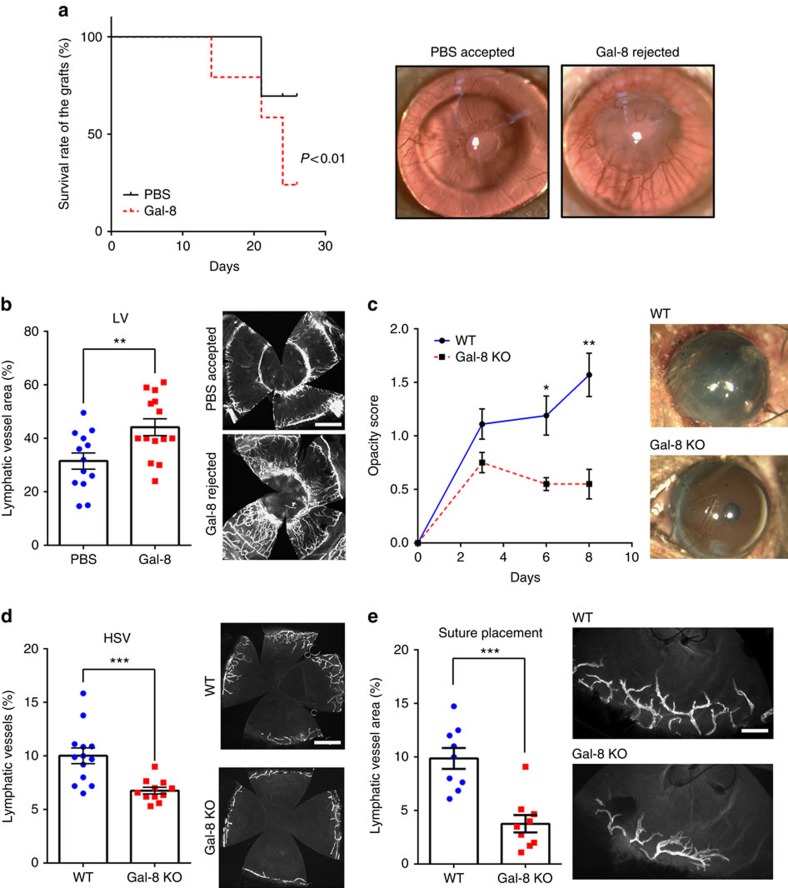
Galectin-8 modulates ocular lymphangiogenesis and associated pathology. (**a**,**b**) Galectin-8 promotes corneal graft rejection. (**a**) BALB/c mice (*N*=48) were transplanted with corneal allografts from C57BL/6 mice. Animals were treated with galectin-8 (10 μg for subconjunctival injection and 50 μg for intraperitoneal injection, *N*=27) or PBS (*N*=21) twice a week beginning postoperative week 1. Grafts were evaluated weekly for signs of rejection by slit-lamp biomicroscopy. Representative photomicrographs of accepted or rejected allografts are shown in the right panel. Kaplan–Meier survival curve demonstrates that galectin-8 promotes allograft rejection. (**b**) On postoperative week 4, PBS-treated corneas (*N*=13) or galectin-8-treated corneas (*N*=14) were harvested and corneal flat mounts were stained with anti-LYVE-1, and lymphatic vessel areas were quantified (left). Representative fluorescence images are shown in the right panel. Data are plotted as mean±s.e.m. and analysed using Log-rank (Mantel–Cox) test (**a**) and Student's *t*-test (**b**). **P<0.01 versus PBS. (**c**,**d**) Lymphangiogenesis and severity of HSV keratitis is reduced in galectin-8 KO mice. WT (*N*=13) and galectin-8 KO (*N*=8) mouse corneas were scarified and infected with 1 × 10^6^ pfu of HSV-1. The progression of HSV keratitis was assessed by corneal opacity scores on day 3, 6 and 8 post infection (**c**). Representative photomicrographs of eyes on day 8 post infection are shown in the right panel. After 8 day post infection, the corneal flat mounts were stained with anti-LYVE-1 and lymphatic vessel areas were quantified (**d**). Representative fluorescence images are shown in the right panel. (**e**) Suture-induced inflammatory lymphangiogenesis is reduced in galectin-8 KO mice. Sutures were placed 2 mm above the limbal vessel in the corneas of WT (*N*=9) and galectin-8 KO (*N*=9) mice. After 7 day post-surgery, the corneal flat mounts were stained with anti-LYVE-1 and lymphatic vessel areas were quantified. Representative fluorescence images are shown in the right panel. Data are plotted as mean±s.e.m. and analysed using Student's *t*-test. *P<0.05, **P<0.01, ***P<0.001 versus PBS (**b**) or WT (**c**,**d**,**e**). Scale bars: 800 μm (**b**,**d**); 200 μm (**e**).

Since lymphangiogenesis contributes to corneal graft rejection^3^,^37^,^38^, we sought to determine if galectin-8-induced lymphangiogenesis promotes graft rejection in a mouse model of corneal transplantation. To test this, we performed allogeneic corneal transplantation (donor, male C57BL/6 mice; recipient, male BALB/c mice; both 10 weeks old) and treated the recipient mice with recombinant galectin-8 or PBS (subconjunctivally and intraperitoneally, twice a week beginning at day 7). On postoperative week 4, graft survival rate was markedly decreased in galectin-8-treated mice compared with PBS-treated mice (survival rates: PBS, 66.7%, 14 out of 21; galectin-8, 18.5%, 5 out of 27) (Fig. 3a). Excised corneas from each group were assessed for the extent of corneal lymphangiogenesis on postoperative week 4. The extent of lymphangiogenesis in galectin-8-treated corneas was significantly higher than that of control corneas (Fig. 3b). These results support the notion that galectin-8-induced lymphangiogenesis promotes graft rejection.

Next, to determine the role of endogenous galectin-8 in lymphangiogenesis *in vivo*, we utilized two different mouse models of pathological lymphangiogenesis in WT and galectin-8 KO mice. HSV-1 keratitis is one of the most common ocular infections. It is characterized by recurrent episodes and is the leading cause of infectious corneal blindness in the developed countries^39^. HSV-1 infection drives corneal lymphangiogenesis^7^ and inhibiting lymphangiogenesis is thought to be of potential therapeutic value to alleviate corneal pathology caused by HSV-1 infection^40^. To determine if endogenous galectin-8 plays a role in HSV-induced pathology and lymphangiogenesis, we infected mouse corneas of WT and galectin-8 KO mice with a clinical isolate of HSV-1. As expected, on day 8 post infection, HSV-1 infection caused corneal opacification (Fig. 3c) and induced corneal lymphangiogenesis (Fig. 3d). In contrast, galectin-8 deficiency not only ameliorated corneal opacity (Fig. 3c) but also reduced corneal lymphangiogenesis (Fig. 3d), suggesting that galectin-8 is involved in the pathogenesis of HSV keratitis, at least partly, by regulating pathological lymphangiogenesis.

Suture placement in the mouse cornea is a commonly used technique to determine inflammatory lymphangiogenesis in the setting of sterile condition (in contrast to infectious agent-induced lymphangiogenesis)^41^,^42^. Clinical relevance of the method stems from the fact that suture placement is routinely used in many corneal surgeries. In this model also, the extent of suture-induced lymphangiogenesis was reduced in the galectin-8 KO mice (Fig. 3e). Together, these findings conclusively demonstrate that galectin-8 is required for robust pathological lymphangiogenesis.

### Effect of galectin-8 on VEGF-C-induced LEC sprouting and lymphangiogenesis

**Figure 4:**
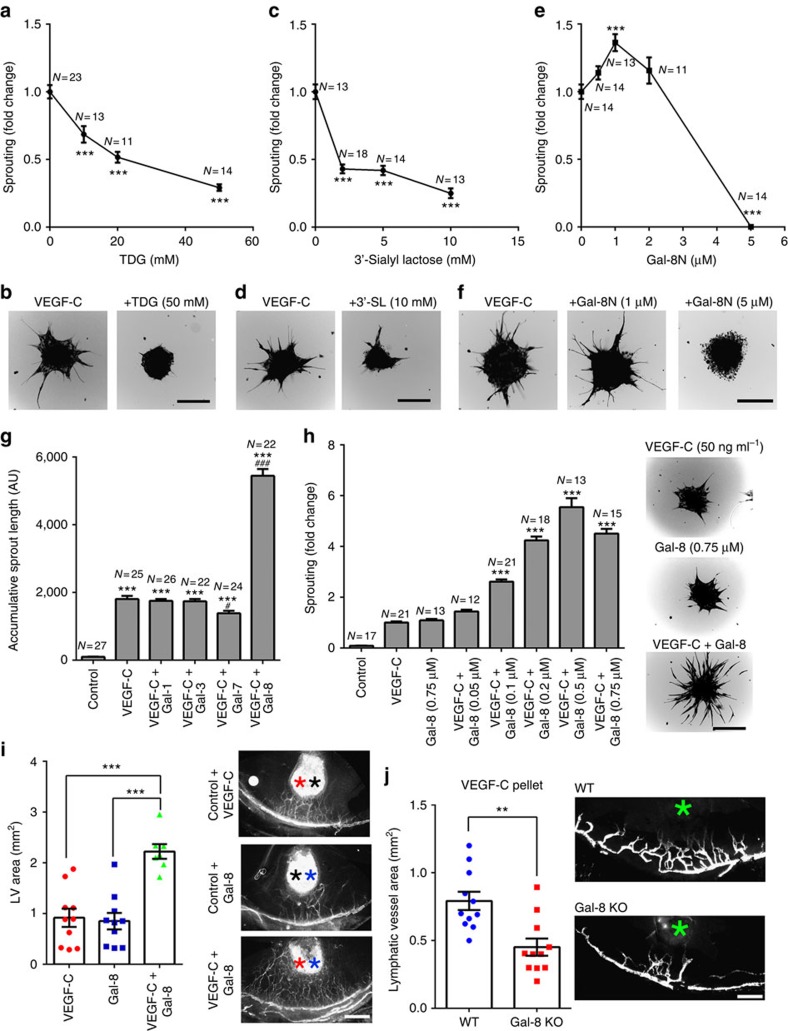
Galectin-8 modulates VEGF-C-induced lymphangiogenesis. (**a**–**f**) VEGF-C-induced LEC sprouting is inhibited by inhibitors of galectin-8. LEC spheroids were stimulated with VEGF-C (50 ng ml^−1^) in the presence or absence of TDG (**a**,**b**), 3′-SL (**c**,**d**) or Gal-8N (**e**,**f**). After 24 h, accumulated sprout lengths were quantified. A value of 1.0 was assigned to the sprout length of VEGF-C-treated LEC spheroids. The values for inhibitor-treated groups are expressed as a change with respect to VEGF-C-treated LEC spheroids. Representative images are shown in **b**,**d**,**f**. (**g**) Galectin-8, but not galectins-1, 3 or 7, has a synergistic effect on VEGF-C-induced LEC sprouting. (**h**) Synergistic effect of galectin-8 on VEGF-C-induced LEC sprouting is dose-dependent. The LEC spheroids were treated with VEGF-C (50 ng ml^−1^), in the presence or absence of galectin-8. A value of 1.0 was assigned to the sprout length of VEGF-C-treated cells. The values for all other groups are expressed as a change with respect to VEGF-C-treated LEC spheroids. Representative fluorescent images are shown in the right panel. (**i**) Galectin-8 markedly enhances VEGF-C-induced lymphangiogenesis *in vivo*. Two separate pellets, one containing VEGF-C and the other galectin-8, were implanted in the corneas of *Prox1*-EGFP reporter mice. One week after surgery, the vessel area was calculated as described in the text. Representative fluorescent images are shown in the right panel. Black asterisk, control pellet; red asterisk, VEGF-C pellet; blue asterisk, galectin-8 pellet. (**j**) VEGF-C-induced lymphangiogenesis is reduced in galectin-8 KO mice. VEGF-C pellets (160 ng) were implanted into WT (*N*=11) and galectin-8 KO (*N*=11) mice. After 7 day post implantation, the corneal flat mounts were stained with anti-LYVE-1 and lymphatic vessel areas were quantified. Asterisks indicate VEGF-C pellets. Data are plotted as mean±s.e.m. and analysed using one-way ANOVA (**g**,**h**,**i**) and Student's *t*-test (**a**,**c**,**e**,**j**). ***P<0.001 versus control (**g**), VEGF-C (**a**,**c**,**e**,**h**). ^#^*P*<0.05, ^###^*P*<0.001 versus VEGF-C (**g**). ANOVA, analysis of variance; TDG: thiodigalactoside; 3′-SL, 3′-sialyllactose; Gal-8N, N-terminal CRD. The results are representative of three or more independent experiments. Scale bars: 100 μm (**b**,**d**,**f**,**h**); 400 μm (**i**); and 200 μm (**j**).

To gain mechanistic insight on galectin-8-mediated LEC sprouting and lymphangiogenesis, first we tested whether the lectin influences the function of a well-known lymphangiogenic molecule, VEGF-C. VEGF-C-induced LEC sprouting was inhibited by galectin-8 inhibitors: TDG (a pan inhibitor of galectins, Fig. 4a,b), 3′-sialyl lactose (the high affinity ligand of the N-CRD of galectin-8, Fig. 4c,d), and by Gal-8N (the dominant negative inhibitor of galectin-8; Fig. 4e,f). These data establish the critical role of galectin-8-dependent carbohydrate-mediated recognition in VEGF-C-induced LEC sprouting.

To further determine the effect of galectin-8 on VEGF-C-induced LEC sprouting, we tested the effect of exogenous galectin-8 on VEGF-C-induced LEC sprouting. Galectin-8, but not galectins-1, 3 and 7, markedly enhanced VEGF-C-induced LEC sprouting (Fig. 4g). In addition, at 0.75 μM of galectin-8, VEGF-C-induced LEC sprouting was five times higher than that seen by VEGF-C alone or galectin-8 alone (Fig. 4h), indicating that galectin-8 has a synergistic effect on VEGF-C-induced LEC sprouting. In the *in vivo* micropocket assays also, galectin-8 collaboratively augmented VEGF-C-induced lymphangiogenesis (Fig. 4i). Moreover, in galectin-8 KO mice, the extent of VEGF-C-induced lymphangiogenesis was significantly reduced (Fig. 4j).

### Galectin-8- and VEGF-C-induced LEC sprouting is dependent on PDPN

It has been reported that cell surface receptor clustering by the galectin-glycan lattices increases the magnitude or duration of signalling from the cell surface^43^,^44^. Therefore, in an effort to characterize the mechanism by which galectin-8 modulates VEGF-C-induced lymphangiogenesis, we first conducted a study to determine whether the lectin modulates VEGFR-3, the predominant VEGF-C receptor. This study revealed that VEGFR-3, but not VEGF-C, is a galectin-8-binding protein (Supplementary Fig. 4a,b), and that galectin-8 clusters VEGFR-3 on cell surface (Supplementary Fig. 4c). However, surprisingly, knockdown of VEGFR-3 had little effect on galectin-8-induced LEC sprouting (Supplementary Fig. 5a–c), suggesting that molecules besides VEGFR-3 are involved in galectin-8-induced LEC sprouting. Therefore, we sought to determine whether galectin-8-mediated LEC sprouting involves other receptors for VEGF-C. In this respect, it is known that VEGFR-2, which also binds VEGF-C, is not involved in VEGF-C-induced sprouting^45^,^46^. Our siRNA knockdown and/or antibody blocking studies revealed that several other known receptors of VEGF-C including neuropilin-2 and integrin α9β1 (refs 47, 48) are not possible targets of galectin-8 (Supplementary Figs 5d–f and 6).

**Figure 5:**
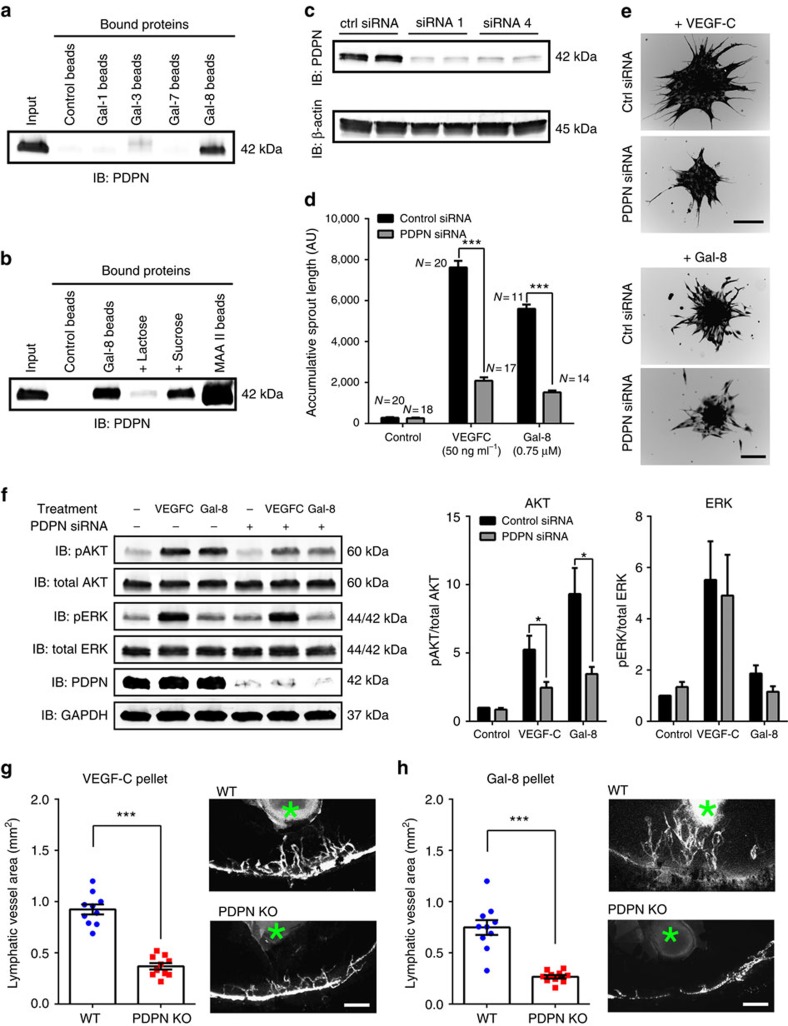
Galectin-8- and VEGF-C-induced lymphangiogenesis is dependent on PDPN. (**a**) PDPN binds to galectin-8, but not to galectins-1, 3 or 7. (**b**) Galectin-8 binds to PDPN in a carbohydrate-dependent manner. LEC lysates were incubated with galectin-8 conjugated to agarose beads in the presence or absence of lactose or sucrose (100 mM), and MAA II conjugated to agarose beads. Bound proteins were examined along with total cell lysates (input) by western blot using anti-PDPN. (**c**) PDPN knockdown: primary LECs were transfected with control (mock) or two siRNA that targeted different regions of PDPN. Knockdown efficiency was assessed by western blot using anti-PDPN and anti-β-actin antibodies. (**d**–**e**) PDPN knockdown inhibits VEGF-C- as well as galectin-8-induced LEC sprouting. Spheroids prepared using primary LECs transfected with control or pooled PDPN siRNA were treated with galectin-8 (0.75 μM) or VEGF-C (50 ng ml^−1^). After 24 h, accumulated sprout lengths were quantified (**d**). Representative images of the sprouts are shown in the right panel. (**f**) PDPN knockdown significantly decreases galectin-8-induced as well as VEGF-C-induced activation of AKT but not ERK. Primary LECs were transfected with control or PDPN siRNA. The cells were serum-starved and treated with galectin-8 (0.5 μM) or VEGF-C (50 ng ml^−1^, positive control) for 30 min. Electrophoresis blots of cell lysates were probed with indicated antibodies. Quantification of fluorescence intensity of western blots (*N*=5) is shown in right panels. (**g**,**h**) PDPN deficiency diminishes VEGF-C- and galectin-8-induced lymphangiogenesis. VEGF-C pellets (160 ng) (*N*=10) (**g**) and galectin-8 pellets (160 ng) (*N*=9) (**h**) were implanted into WT and PDPN inducible KO mice. After 7 days post implantation, the corneal flat mounts were stained anti-LYVE-1 and lymphatic vessel areas were quantified as described in Methods. Representative fluorescence images from each group are shown. Asterisks indicate pellets. Data are plotted as mean±s.e.m. and analysed using Student's *t*-test. *P<0.05, **P<0.01, ***P<0.001 versus corresponding control or WT mice. Scale bars: 100 μm (**e**); and 200 μm (**g**,**h**).

Next we performed studies to determine whether galectin-8-induced LEC sprouting is dependent on PDPN. PDPN is thought to play a role in lymphangiogenesis^19^,^20^. However, the role of galectin-8 in the modulation of PDPN has thus far not been fully investigated and virtually nothing is known about the role of PDPN in VEGF-C-induced lymphangiogenesis. Here, we first showed that PDPN expressed in LECs interacts with galectin-8, but not galectins-1, 3 or 7 (Fig. 5a), and the binding of PDPN to galectin-8 was carbohydrate-dependent (Fig. 5b). In addition, PDPN expressed in LECs contains α2,3-sialylated glycans (Fig. 5b). Removal of α2,3-sialylated glycans by treatment with α2-3 neuraminidase abrogated the interaction of PDPN and galectin-8, suggesting that galectin-8 binds α2,3-sialylated glycans of PDPN (Supplementary Fig. 6 and Supplementary Methods). Secondly, to determine whether PDPN plays a role in galectin-8- and/or VEGF-C-induced LEC sprouting, spheroids prepared using primary LECs transfected with control or PDPN siRNA were treated with galectin-8 or VEGF-C. The expression of PDPN was reduced by 82% in the siRNA transfected LECs (Fig. 5c). PDPN knockdown not only markedly inhibited galectin-8-induced LEC sprouting, but also substantially reduced VEGF-C-induced LEC sprouting (Fig. 5d,e). Thirdly, PDPN knockdown in LECs substantially reduced galectin-8- and VEGF-C-induced activation of AKT but not ERK (Fig. 5f), suggesting that PDPN modulates galectin-8- and VEGF-C-induced LEC sprouting largely by activation of AKT pathway.

Lastly, to determine whether PDPN plays a role in galectin-8- and/or VEGF-C-induced lymphangiogenesis *in vivo*, we used mice with tamoxifen-inducible global deletion of PDPN (*Pdpn*^*f/f*^*;CagCre*)^49^ to perform the corneal micropocket assays. As expected, VEGF-C pellets markedly induced both haemangiogenesis and lymphangiogenesis in WT mice. In contrast, VEGF-C-induced lymphangiogenesis was significantly reduced in the PDPN-deficient mice (Fig. 5g). Likewise, galectin-8-induced lymphangiogenesis (Fig. 5h) was reduced in PDPN-deficient mice. Together, the data suggest that PDPN is a key player in, not only galectin-8-induced lymphangiogenesis, but also VEGF-C-induced lymphangiogenesis.

### Interplay among integrins, galectin-8 and PDPN

**Figure 6:**
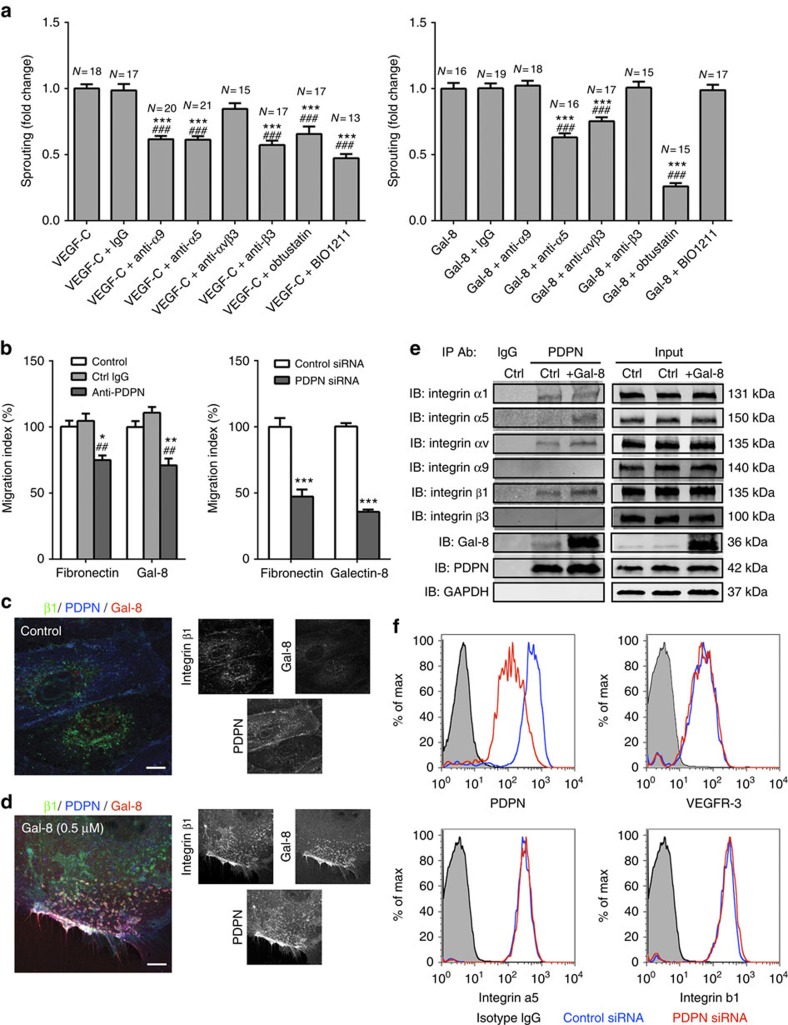
Integrins regulate galectin-8-mediated LEC sprouting and migration. (**a**) α1β1 and α5β1 inhibition reduces both VEGF-C- and galectin-8-induced LEC sprouting. LEC spheroids were stimulated with VEGF-C or galectin-8 in the presence or absence of control IgG, blocking antibodies and peptides. Obtustatin: specific for integrin α1β1; BIO1211: specific for integrin α4β1. A value of 1.0 was assigned to the sprout length of VEGF-C or galectin-8-treated LEC spheroids. (**b**) Matrix-mediated LEC migration is dependent on PDPN. LECs incubated with anti-PDPN Ab (left), or transfected with PDPN siRNA (right) were seeded into the upper chamber of Transwell inserts. The lower side of the insert membrane was coated with fibronectin or galectin-8. After 2 h incubation, LECs migrated to lower side of the membrane were counted. Results are expressed as % change where control is set as 100%. (**c**,**d**) Galectin-8 clusters integrin β1 and PDPN on cell surface. LECs were treated with or without galectin-8 for 15 min, fixed without permeabilization, stained with antibodies to integrin β1 (green), PDPN (blue) and galectin-8 (red), and examined by confocal microscopy. (**c**) Control LECs. (**d**) Exogenous galectin-8 sequesters integrin β1 and PDPN (white spots in the merged image) after galectin-8 treatment. Scale bar, 7.5 μm. (**e**) Integrins α5 and β1 interact with PDPN in a galectin-8-dependent manner. LECs were incubated with or without galectin-8 (0.5 μM) for 15 min at 37 °C. Cell lysates were immunoprecipitated with control or anti-PDPN antibodies, and were processed for western blotting using antibodies indicated. (**f**) Cell surface expression of integrins α5, β1 and VEGFR-3 remains similar in PDPN knockdown cells. Flow cytometry analysis was used to assess cell surface expression levels of PDPN, VEGFR-3, integrins β1 and α5 in LECs transfected with control siRNA (blue line) or PDPN siRNA (red line). Black lines: cells stained with isotype IgG. Data are plotted as mean±s.e.m. and analysed using one-way ANOVA (**a**,**b**) and Student's *t*-test (**b**). ***P<0.001 versus VEGF-C or Gal-8 (**a**); ^###^*P*<0.001 versus VEGF-C or Gal-8+control IgG (a). *P<0.05, **P<0.01 versus control IgG (**b**); ***P<0.001 versus control siRNA (**b**); ^##^*P*<0.01 versus control IgG (b). ANOVA, analysis of variance.

In addition to α9β1 integrin, several other integrins including α1β1, α4β1 and α5β1 are involved in the process of lymphangiogenesis^24^ and the interplay between integrin β1 and VEGFR-3 has been reported^25^,^26^. Therefore, to determine whether galectin-8–PDPN-induced LEC sprouting involves specific integrins; first, LEC spheroids were treated with galectin-8 or VEGF-C in the presence or absence of blocking antibodies and peptides against a panel of integrins. In this study, only blocking of integrins α1β1 (by obtustatin) and α5β1 (by the neutralizing antibody) inhibited both VEGF-C- and galectin-8-induced LEC sprouting (Fig. 6a). Secondly, to determine whether PDPN indirectly regulates the functions of VEGF-C/VEGFR-3 through controlling the function of integrins α1β1 and α5β1 in galectin-8-dependent manner, we performed studies to determine whether: (i) PDPN inhibition attenuates matrix-mediated LEC migration; (ii) PDPN interacts with integrins α5β1 in a galectin-8-dependent manner; and (iii) knockdown PDPN impedes integrin-mediated signalling cascades. In this study, blocking the function of PDPN by antibodies as well as siRNA knockdown attenuated both fibronectin- and galectin-8- promoted cell migration (Fig. 6b). These data in conjunction with a published study^15^ showing that PDPN-Fc inhibits type I collagen-mediated LEC migration, suggest that PDPN is involved in not only galectin-8 but also fibronectin- and type I collagen-mediated LEC migration, a process in which integrins are well-known to play a key role. To assess the galectin-8-dependent interaction between integrin β1 and PDPN, primary LECs were treated with galectin-8 for 15 min, fixed without permeabilization, stained with antibodies against integrin β1, PDPN and galectin-8, and examined by confocal microscopy. In untreated control cells, galectin-8, integrin β1 and PDPN were homogenously distributed all over the LECs (Fig. 6c). Since we showed that galectin-8 was upregulated in inflamed corneas (Fig. 1), we added the exogenous galectin-8 to see whether galectin-8 changes the distribution of PDPN and/or integrin β1 on LECs. Addition of galectin-8 caused dramatic redistribution and clustering of PDPN and integrin β1 on LEC plasma membrane (Fig. 6d). To more directly assess the association between integrins and PDPN, lysates from untreated or galectin-8-treated LECs were incubated with anti-PDPN antibody, and immunoprecipitated proteins were examined by western blotting using antibodies against specific integrins, galectin-8 and PDPN. In untreated cell lysates, immunoprecipitation with anti-PDPN co-immunoprecipitated endogenous galectin-8 and specific integrins (α1, α5, αv, β1, but not α9 or β3), indicating that PDPN interacted with endogenous galectin-8 and the association between PDPN and specific integrins (α1, α5, αv and β1) was constitutive (Fig. 6e). When cells were treated with exogenous galectin-8, there was an increased association between PDPN and integrins α5, αv, β1, while the association between PDPN and integrin α1 remained similar (Fig. 6e).

Next, to assess the role of PDPN on integrin-mediated signalling, LECs transfected with control or PDPN siRNA were seeded on collagen I- or fibronectin-coated wells and allowed to adhere for 15 min at 37 °C. Cell lysates from attached cells on collagen I or fibronectin were analysed with western blotting using phospho-specific integrin and focal adhesion kinase (FAK) antibodies. Phosphorylation of integrin β1 was reduced in PDPN knockdown cells (Supplementary Fig. 7), suggesting that the activation of integrin β1 is reduced in the absence of PDPN. Also, phosphorylation of FAK was decreased in the PDPN knockdown cells seeded on collagen I- but not fibronectin-coated wells (Supplementary Fig. 7), whereas phosphorylation of ERK was markedly reduced in the PDPN knockdown cells seeded on both matrix proteins (Supplementary Fig. 7). To eliminate the possibility that the loss of integrin activation and signalling in the PDPN knockdown cells was not due to altered expression and cellular distribution of integrins and VEGFR-3, we performed studies to determine whether PDPN knockdown alters the cell surface expression of integrins and/or VEGFR-3, using two different approaches: cell surface biotinylation and flow cytometry analysis. In the cell surface biotinylation approach, GAPDH and Prox1 were not detected in the streptavidin pulldown cell lysates, and cell surface PDPN was markedly reduced in the PDPN knockdown cells (Supplementary Fig. 8 and Supplementary Methods). Expression of cell surface VEGFR-3, integrins α5 and β1 was similar in the PDPN knockdown cells and control cells (Supplementary Fig. 8). Similarly, in the flow cytometry analysis approach, cell surface expression of PDPN was reduced, whereas that of VEGFR-3, integrins α5 and β1 did not change in the PDPN knockdown cells (Fig. 6f). Thus, PDPN deficiency does not change the cell surface expression of integrins α5/β1 and VEGFR-3 of LECs. Furthermore, transcriptome analysis of PDPN knockdown and control cells revealed that the coefficient of determination (R^2^) of the linear curve fitting was more than 0.9 (Supplementary Fig. 9, Supplementary Data 1 and Supplementary Methods), indicating that most genes are not differentially expressed. Taken together, these data lead us to conclude that PDPN regulates the functions of integrin β1 complexes, specifically of integrins α1β1 and α5β1 in LECs, and that this function is galectin-8-dependent.

### Galectin-8 inhibitors decrease inflammatory lymphangiogenesis

**Figure 7:**
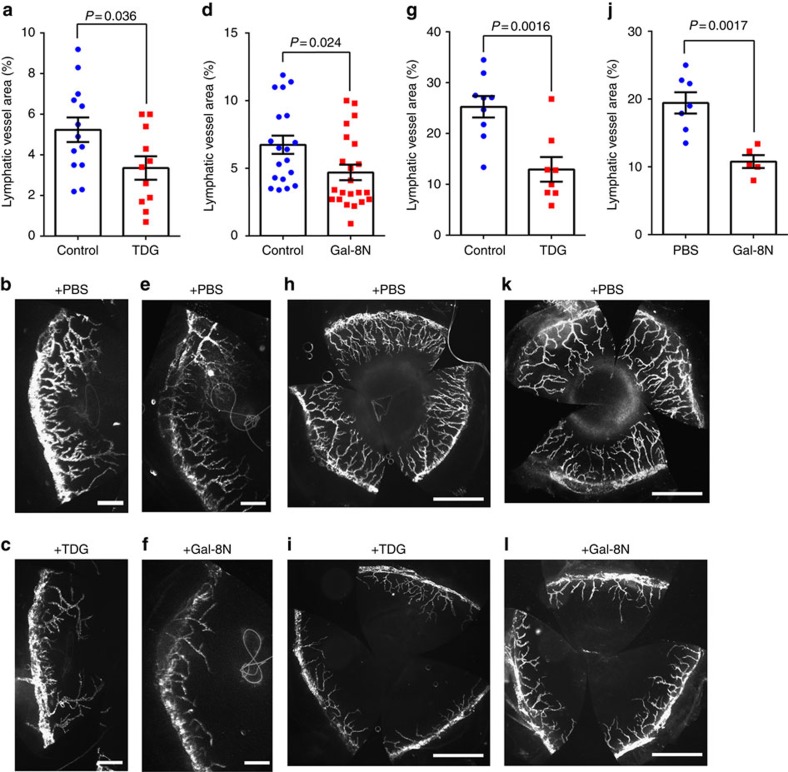
Galectin inhibitors markedly decrease inflammatory lymphangiogenesis *in vivo*. (**a**–**f**) Sutures were placed ∼2 mm above the limbic vessel in the corneas of the *Prox1*-EGFP reporter mice (*N*=12 or more). (**g**–**l**) Silver nitrate cautery was introduced in the centre of the corneas of the *Prox1*-EGFP reporter mice (*N*=5 or more). The animals were treated with TDG (200 mM in 10 μl, a pan inhibitor of galectins) (**c**,**i**), or Gal-8N (15 μg in 10 μL, a dominant negative inhibitor of galectin-8) (**f**,**l**) by local subconjunctival injections on days 0, 2, 4 and 6 post-surgery. At the end of the treatment period, lymphatic vessel areas were quantified (**a**,**d**,**g**,**j**). Representative images are shown in the bottom panels. Data are plotted as mean±s.e.m. and analysed using Student's *t*-test. The results are representative of two independent experiments. Scale bar: 200 μm (**b**,**c**,**e**,**f**); and 1 mm (**h**,**i**,**k**,**l**).

To determine whether galectins can be targeted to control lymphangiogenesis, two *in vivo* models of lymphangiogenesis were used. After suture placement and AgNO_3_ cauterization in the corneas of *Prox1*-EGFP reporter mice to induce inflammation, the mice were treated with TDG (200 mM, a pan inhibitor of galectins) or Gal-8N (15 μg, the dominant negative inhibitor of galectin-8) by subconjunctival injections on days 0, 2, 4 and 6 post surgery. At the end of the treatment period, lymphatic vessel areas were quantified. Treatment with both TDG (Fig. 7a,c,g,i) as well as Gal-8N (Fig. 7d,f,j,l) significantly suppressed suture- and cautery-induced corneal lymphangiogenesis. These data suggest a promising new mechanism for the modulation of pathological lymphangiogenesis by targeting galectin-8.

## Discussion

We demonstrate here that galectin-8 is highly upregulated in pathological corneas and plays a critical role in the process of lymphangiogenesis. The striking finding that several other members of galectin family including galectins-1 and 3, which are known to promote hemangiogenesis, did not promote LEC sprouting suggests that galectin-8-mediated LEC sprouting involves the affinity of N-CRD of galectin-8 for 3′-sialylated galactosides that is unique among animal galectins^11^,^12^,^13^. In support of this notion, specific inhibition of the N-CRD of galectin-8 with 3′-SL reduced LEC sprouting and a galectin-8 mutant, Gal-8Q47A, which has lost its ability to bind to α2,3-sialyl glycans, did not promote LEC sprouting. Together, these data establish that galectin-8 promotes LEC sprouting in a carbohydrate-dependent manner and that N-CRD of galectin-8 was directly involved in the stimulatory effect of galectin-8 on LEC sprouting.

A major finding of the current study is that galectin-8 modulates VEGF-C-mediated lymphangiogenesis. Our studies show that Gal-8N (the dominant-negative inhibitor of galectin-8) and 3′-SL (a competing disaccharide) ameliorated VEGF-C-induced LEC sprouting. Furthermore, in the *in vivo* corneal micropocket assay, the extent of lymphangiogenesis induced by VEGF-C was significantly less in galectin-8 KO mice compared with the WT mice. To our knowledge, this is the first demonstration of a defect in lymphangiogenic response of galectin-8 KO mice. In addition, exogenous galectin-8 markedly enhanced VEGF-C-induced LEC sprouting *in vitro* and lymphangiogenesis *in vivo* in a carbohydrate-dependent manner. Together, these data conclusively establish that galectin-8 significantly influences VEGF-C-mediated lymphangiogenesis. Of note, the inhibitory effect of Gal-8N on VEGF-C-induced LEC sprouting is bell-shaped, which is similar to several other anti-(lymph)angiogenic molecules such as RGD-mimetic integrin inhibitors (ref. 50). Not surprisingly, much higher concentration of galectin-8 (0.75 μM), compared with VEGF-C (2.38 nM), was required to produce equivalent LEC sprouting. This is because generally, the affinity of CRD of galectins towards their glycan ligands is lower (dissociation constant:∼μM) compared with typical protein−protein interaction (dissociation constant:∼10 nM)^51^. Despite the weak affinity of their CRD, galectins achieve a stable interaction with their ligands due to their multivalency that results in overall high avidity^52^. Therefore, even if the affinity of one galectin-8 molecule for one PDPN molecule is weak, the overall high avidity is able to activate lymphangiogenesis pathway.

Our findings that PDPN binds to galectin-8 in a carbohydrate-dependent manner, that it contains the high affinity glycans of galectin-8 (α2,3-sialylated glycans), that galectin-8 clusters PDPN on cell surface, and that unlike the knockdown of VEGFR-3, knockdown of PDPN abrogates galectin-8-induced LEC sprouting suggest that PDPN is a key player in the mechanism of galectin-8-induced LEC sprouting. Another major finding of the current study is that PDPN plays a critical role in VEGF-C-mediated lymphangiogenesis. Thus far, VEGF-C- and PDPN-mediated pathways have been independently shown to promote lymphangiogenesis, but the relationship in the molecular mechanism of the two pathways has not been demonstrated. Overall, our findings suggest that a galectin-8-dependent cross-talk among VEGF-C, PDPN, and integrin pathways plays a critical role in lymphangiogenesis. This is an important conceptual advance in the understanding of the molecular mechanism of a well-known VEGF-C lymphangiogenic pathway.

**Figure 8:**
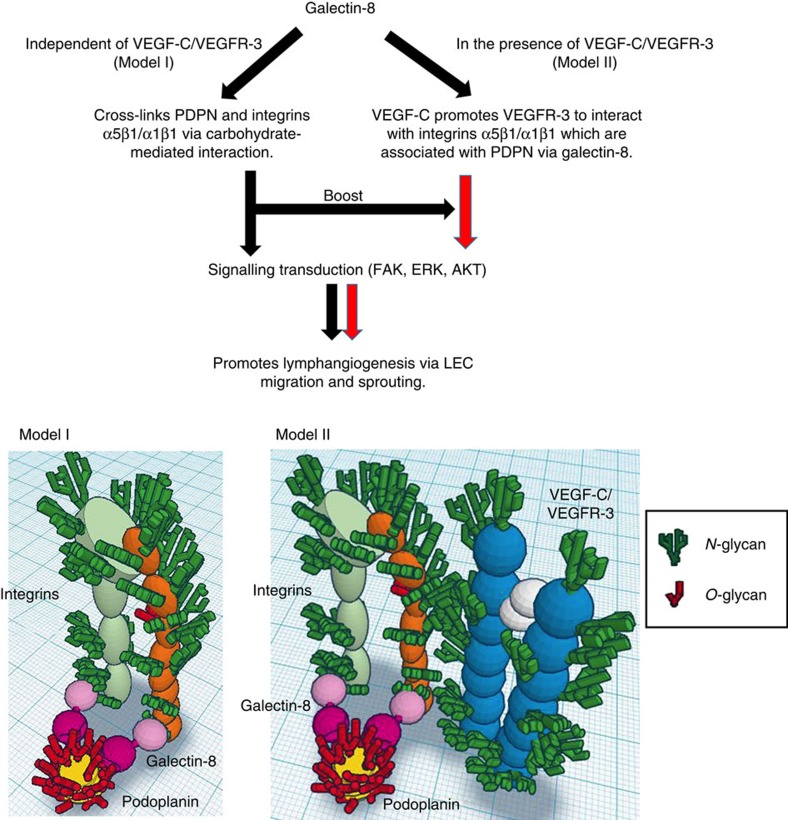
Schematic representation and proposed models. According to model I, galectin-8 crosslinks and clusters integrins α1β1/α5β1 and PDPN on the cell surface. The clustering activates lymphangiogenic signalling pathways that modulate events such as endothelial cell migration and sprouting without the involvement of VEGFR-3. This model is supported by current studies showing that (i) VEGFR-3 is dispensable in galectin-8-mediated lymphangiogenesis, (ii) galectin-8-mediated lymphangiogenesis is dependent on PDPN and integrins α1β1/ α5β1, and (iii) galectin-8 treatment increases the interaction of PDPN and integrin β1. In the presence of VEGFR-3 (Model II), PDPN−galectin-8−integrin interactions substantially increase the magnitude of lymphangiogenic pathway by potentiating the VEGF-C/VEGFR-3 signalling. This model is supported by our findings that galectin-8 potentiates VEGF-C-induced lymphangiogenesis *in vitro* and *in vivo*, that galectin-8 inhibitors attenuate VEGF-C-induced lymphangiogenesis *in vitro* as well as *in vivo*, and that Gal-8-induced lymphangiogenesis is reduced by α1β1 and α5β1 inhibitors. Note that only extracellular domains of glycoproteins are shown.

Schematic representation of mechanistic aspects of galectin-8-induced lymphangiogenesis is shown in Supplementary Fig. 10. Our studies revealed that VEGFR-3 is a galectin-8-binding protein (Supplementary Fig. 4a,b), and galectin-8 clusters and retains VEGFR-3 on cell surface (Supplementary Fig. 4c). Despite this, VEGFR-3 knockdown did not inhibit galectin-8-induced sprouting (Supplementary Fig. 5). This suggests that although VEGF-C-induced LEC sprouting is dependent on extracellular galectin-8, the lectin has the capacity to promote LEC sprouting independently of VEGFR-3, and VEGFR-3 may be a pseudoreceptor for galectin-8. We reason that inhibiting galectin-8 attenuates VEGF-C-mediated signalling because the function of integrins α1β1 and α5β1, rather than VEGFR-3, is inhibited by galectin-8 inhibitors. We propose that galectin-8 has a unique dual-faceted mechanism of action to promote lymphangiogenesis, where galectin-8-mediated interactions between lymphangiogenic integrins (α1β1/α5β1) and PDPN, are sufficient to activate the integrins and trigger the process of lymphangiogenesis without the involvement of VEGFR-3 (model I, Fig. 8), but in the presence of VEGF-C/VEGFR-3, PDPN-galectin-8-integrin interactions substantially increase the magnitude of lymphangiogenic pathway by potentiating the VEGF-C/VEGFR-3 signalling (model II, Fig. 8). Model I is supported by current studies showing that (i) VEGFR-3 is dispensable in galectin-8-mediated LEC sprouting; (ii) galectin-8-mediated lymphangiogenesis is dependent on PDPN and integrins α1β1/α5β1; and (iii) galectin-8 treatment increases the interaction of PDPN and integrin β1 (Supplementary Fig. 9). Model II is supported by our findings that galectin-8 potentiates VEGF-C-induced lymphangiogenesis, that galectin-8 inhibitors attenuate VEGF-C-induced lymphangiogenesis, and that galectin-8-induced LEC sprouting is reduced by α1β1 and α5β1 inhibitors (Supplementary Fig. 9).

Various studies have demonstrated the pathological contribution of lymphangiogenesis to diseases of the eye^2^,^3^,^4^,^5^,^6^,^7^. Specifically, corneal lymphatics play a vital role in the pathogenesis of graft rejection^3^,^37^,^38^,^53^,^54^,^55^, herpetic keratitis^7^,^56^, dry eye disease^4^, ocular allergy^5^ and wound healing^57^. In the present study, we demonstrated that: (i) a dominant negative inhibitor of galectin-8 as well as the pan inhibitor of galectins dampen lymphangiogenesis; (ii) in the mouse model of corneal allogeneic transplantation, galectin-8-induced lymphangiogenesis is associated with an increase in corneal graft rejection; and that (iii) in the mouse model of HSV keratitis, corneal pathology and lymphangiogenesis is ameliorated in galectin-8 knockout mice. These findings have broad implications for developing novel therapeutic strategies for conditions resulting from pathological lymphangiogenesis in both ocular diseases mentioned above as well as nonocular diseases such as cancer metastasis and solid organ transplant rejection. Indeed, the avascular cornea serves as an excellent *in vivo* model to study mechanisms of hem- and lymph-angiogenesis that are also relevant to nonocular diseases. For example, based on the findings that lymphatics in the cornea promotes graft rejection, subsequent studies reported that lymphangiogenesis also occurs in solid organ grafting, such as renal and cardiac transplantation^58^,^59^,^60^. Undeniably, corneal micropocket assays have proven to be extremely valuable in revealing the mechanism of angiogenesis in cancer and many other nonocular tissues. In conclusion, our study offers a new perspective on how glycans of the cell surface receptors can be exploited to understand and modulate the process of lymphangiogenesis.

## Methods

### Study approval

All animal procedures were approved by the Institutional Animal Care and Use Committee at Tufts University and were performed in accordance with the regulations of Association for Research in Vision and Ophthalmology Resolution on the Use of Animals in Vision Research and recommendations of the National Institutes of Health Guide for the Care and Use of Laboratory Animals. Tufts University Institutional Review Board/ethics committee approval was obtained for human specimen acquirement for this study.

### Mice

Lymphatic-specific *Prox1*-EGFP reporter mice (FVB background)^61^ were purchased from Mutant Mouse Regional Resource Centers, FVB/NCrl mice were purchased from Charles River Laboratories, and C57BL/6 mice were purchased from Jackson Laboratory. Mice with inducible deletion of PDPN (*Pdpn*^*f/f*^*;CagCre*) and WT littermates (*Pdpn*^*f/w*^*;CagCre*) in mixed background (C57BL/6 and 129/Sv) were generated as previously described^49^. PDPN deletion was accomplished by administering tamoxifen orally (20 μg each day) from P1 to P6. After weaning, the mice were orally administered 1 mg tamoxifen weekly. The *Lgals8* KO mouse strain used for this study was created from embryonic stem cell clone (14305A-F8), obtained from the KOMP Repository (www.komp.org) and generated by Regeneron Pharmaceuticals, Inc^62^. The *Lgals8* coding region deletion was achieved by *LacZ* (bacterial β-galactosidase) reporter gene replacement of chromosome 13 from 12,440,786 (deletion start) to 12,459,212 (deletion end). Details of the primer sequences and predicted PCR products are available at the Velocigene website (www.velocigene.com/komp/detail/14305). The galectin-8 null status of the KO mice is confirmed by western blotting (Supplementary Fig. 11). The *Lgals8* KO mice have no obvious defects in lymphatic vessel development examined by gross morphological analysis.

### Expression and purification of recombinant proteins

All recombinant human galectins were expressed in the *Escherichia coli* expression system. The recombinant galectins were induced by isopropyl β-D-1-thiogalactopyranoside (IPTG) and purified by affinity chromatography on lactosyl-sepharose beads as descried previously^13^,^31^,^63^. Briefly, human galectin-1 cDNA was cloned into NcoI/Hind III cut pQE-60 (Qiagen, eliminating 6-his tag sequence residing between NcoI and Hind III), transformed into TG1 competent cells and plated onto LB agar containing ampicillin (50 μg ml^−1^). Human galectin-3 cDNA was cloned into NcoI/Hind III cut pKK233-2, transformed into BL21 Star (DE3) competent cells and plated onto LB agar containing ampicillin (50 μg ml^−1^). Human *GALECTIN-7* cDNA was cloned into NcoI/Hind III cut pQE-60 (Qiagen, eliminating 6-His tag sequence residing between NcoI and Hind III), transformed M15[pREP4] competent cells (Qiagen) and plated onto LB agar containing ampicillin (50 μg ml^−1^) and kanamycin (30 μg ml^−1^). For expression and purification of galectin-8 tagged with glutathione *S*-transferase (GST) (GST-galectin-8), human galectin-8 cDNA was cloned into *Sal*I–*Not*I cut pGEX-4T-3 expression plasmid (Pharmacia Biotech) that provides the sequence of GST in the 5′-end, transformed competent cells and plated onto LB agar containing ampicillin (100 μg ml^−1^). For expression and purification of galectin-8 and Gal-8N tagged with thioredoxin (Trx) (Trx-galectin-8 and Trx-Gal-8N), human galectin-8 and Gal-8N constructs in BL21 (DE3) Star competent cells were plated onto LB agar containing ampicillin (50 μg ml^−1^). Induction was achieved by adding IPTG at 1, 0.1, 0.5 and 0.2 mM when OD600 is 0.6 to 0.8 for galectins-1, 3, 7 and 8, respectively. Lysis buffers used for bacterial lysis were (a) 20 mM Tris, pH 7.4, 2 mM EDTA, 150 mM NaCl, 4 mM β-mercaptoethanol and 1 mM PMSF (for galectin-1); (b) PBS with 2 mM EDTA and 4 mM β-mercaptoethanol (for galectin-3); (c) 20 mM Tris, pH 8.0, 5 mM EDTA, 1 mM DTT, 10 μg per ml aprotinin and leupeptine, 1 mM PMSF, 10 mM L-cystein, and lysozyme at 1 mg ml^−1^ (for galectin-7); and (d) PBS with lysozyme at 5 mg ml^−1^ and two tablets of complete protease inhibitor cocktail (for galectin-8). After sonication and centrifugation to remove insoluble proteins, supernatants were passed through lactosyl-sepharose beads (Sigma). Bound proteins were eluted with 150 mM lactose in PBS. Lactose was then removed by dialysis against 5% glycerol in PBS at least four times. Endotoxin was removed by Detoxi-Gel endotoxin removing gel (Thermo Scientific) and endotoxin levels were detected by ToxinSensor chromogenic LAL endotoxin assay kit (Genscript). Endotoxin levels of all recombinant galectins used in this study were <0.1 EU μg^−1^. Before use, each preparation of galectin was also tested for carbohydrate-binding activity by the red blood cell (RBC) agglutination assay. Tag-free recombinant galectin-8 (35.8 kDa, the predominant isoform of galectin-8) was used in this study except that GST (glutathione S-transferase)-tagged galectin-8 was used in affinity precipitation assays. To remove GST tag from GST-galectin-8, the fusion protein was incubated with human thrombin (Sigma, 2 U of thrombin per 1 mg of fusion protein) at 25 °C for 1 h, followed by incubation with *p*-aminobenzamidine-agarose (Sigma) to remove the added thrombin. To remove Trx tag from Trx-galectin-8, the fusion protein was incubated with enterokinase (BioLabs, 6.4 ng per 1 mg of fusion protein) at 4 °C overnight, followed by incubation with trypsin inhibitor agarose to remove the added enterokinase. Recombinant human mature VEGF-C (21 kDa) expressed in mammalian cells was purchased from Peprotech (catalog number: 100-20C).

### Mouse models of corneal chemical injuries

To determine the expression pattern of galectin-8 in chemical injury, C57BL/6 mice were injured by silver nitrate and sodium hydroxide solution, and frozen sections of the eyes were subjected to immunofluorescence staining using anti-galectin-8 antibody as described above. For silver nitrate cautery, silver nitrate applicators (Grafco) were applied on the central cornea of the right eye for 5 s under a surgical microscope. The corneas were rinsed with 2 ml of PBS, and ophthalmic antibiotics were topically applied to the operated eyes. For alkaline burn, sodium hydroxide solution (1.5 μl of 0.15N) was applied to the central corneas of the right eye of each animal for 1.5 min and was immediately rinsed away with PBS^64^. The corneal and limbal epithelium was removed by an Algerbrush (Ambler surgical, Exton, PA). Ophthalmic antibiotic ointment was topically applied to the operated eyes to prevent infection. At the end of the experiment (day 7 post-silver nitrate cautery and day 14 post-alkaline burn), mouse corneas were harvested and embedded in OCT compound.

### Knockdown of target genes

ON-TARGET plus human VEGFR-3, PDPN and Nrp2 siRNA SMART pool was purchased from GE Dharmacon. Oligonucleotide siRNA duplexes targeting PDPN and AllStars Negative control siRNA were purchased from Qiagen. Hs_PDPN_1 and Hs_T1A-2_7 siRNA were designated as PDPN siRNA1 and siRNA4, respectively. Similar results were obtained using PDPN siRNA from GE Dharmacon and Qiagen. The transfection of siRNA in primary LECs (Lonza) was carried out with the Lipofectamine 2000 reagent (Invitrogen). Briefly, 3 μl of Lipofectamine 2000 in 250 μl of Opti-MEM medium (Invitrogen) and 3 μl of siRNA (20 μM) in 250 μl of Opti-MEM medium were incubated separately for 5 min at 25 °C, and the two mixtures were combined and incubated for an additional 20 min to form Lipofectamine 2000-siRNA complexes. At the end of the incubation period, serum-free Opti-MEM (1.5 ml) was added to each well of a six-well plate and 500 μl aliquots of Lipofectamine 2000-siRNA complex were added to each well. Final concentration of siRNA was 30 nM. After 3 h incubation, media were replaced with complete EGM-2MV medium (Lonza). The same procedure was repeated on the next day and knockdown efficiency was assessed by Western blot after 48 h transfection.

### Galectin-8 immunohistochemistry staining of corneas

Paraffin sections of normal and inflamed corneas were obtained from the archived tissues of the Tufts Medical Center Ophthalmic Pathology Laboratory Tissue sections of: (i) normal human corneas were from eyes which were enucleated due to choroidal melanoma (*N*=2) and uveal malignant melanoma (*N*=2); and (ii) inflamed human corneas were obtained at the time of keratoplasty from patients with failed corneal graft (*N*=6), bacterial keratitis (*N*=4) and *Acanthamoeba* keratitis (*N*=2). Tissue sections of normal and inflamed corneas were processed for immunolocalization of galectin-8 using a procedure we have described earlier^65^. Briefly, paraffin-embedded sections were deparaffinized, rehydrated and incubated with rabbit anti-galectin-8 primary antibody (1:100 in 1% BSA/PBS, 1 h, 37 °C, Novus) and a biotinylated secondary antibody (1:300 in 1% BSA/PBS, 30 min, 37 °C, R&D Systems). Sections were subsequently incubated with HSS-HRP (30 min, 25 °C, R&D Systems), and a DAB/AEC chromogen solution (37 °C, R&D Systems). Images were acquired by EVOS XL Core cell imaging system (Invitrogen).

### Immunofluorescence staining of mouse corneas

To induce inflammation, mouse corneas (8-week-old C57BL/6 mice) were treated with thermal cautery and silver nitrate cautery. For thermal cautery, five light burns were applied to the central cornea of the right eye of each animal using the straight fine tip of a hand-held thermal cauterizer (Fine Science Tools) and ophthalmic antibiotics were topically applied to the operated eyes. The eyes with thermal and silver nitrate cautery were harvested on postsurgery day 1 and day 5, respectively. Contralateral eyes served as normal controls. In addition, rejected mouse allografts on postoperative week 4 were also used. Frozen sections of the eyes (12 μm thick) were fixed with 4% paraformaldehyde/PBS, permeabilized with 0.3% Triton X-100/PBS, blocked with 1% BSA/PBS, and were then incubated at 4 °C overnight with primary antibodies (rabbit anti-galectin-8, NBP1-66520, 1:200, Novus; rat anti-mouse CD31, clone MEC13.3, 1:100, BioLegend; biotinylated goat anti-mouse LYVE-1, BAF2125, 1:100, R&D Systems; goat anti-type I collagen, clone D-13, 1:50, SCBT; biotinylated rat anti-mouse Ly6G, clone 1A8, 1:150, BioLegend), followed by incubation at 25 °C for 1.5 h with appropriate secondary antibodies and fluorophore-conjugated antibodies/streptavidin (Alexa Fluor 488 anti-rabbit IgG, 1:300, Invitrogen; Alexa Fluor 568 anti-rabbit IgG, 1:300, Invitrogen; Alexa Fluor 488 rat anti-mouse F4/80, clone BM8, 1:100, BioLegend; Alexa Fluor 647 rat anti-mouse CD11b, clone M1/70, 1:100, BioLegend; Alexa Fluor 647 rat anti-mouse CD45, clone 30-F11, 1:100, BioLegend; Alexa Fluor 488 rat anti-mouse CD4, clone GK1.5, 1:100, BioLegend; Alexa Fluor 488 streptavidin, 1:300, Jackson ImmunoResearch Labs; Alexa Fluor 594 streptavidin, 1:300, Jackson ImmunoResearch Labs; Alexa Fluor 647 donkey anti-rat IgG, 1:300, Invitrogen). Fluorescence images were acquired by Leica TCS SPE imaging system (Leica).

### Corneal mouse micropocket lymphangiogenesis assay

The corneal micropocket lymphangiogenesis assay was performed as described previously using implants containing a test agent, hydron and sucralfate^66^,^67^. Test agents included full-length galectin-8 (40–320 ng per pellet) and VEGF-C (160 ng per pellet). Implants containing hydron and sucralfate alone served as negative controls. The mice were anaesthetized by intraperitoneal injection of a cocktail of ketamine (90–120 mg kg^−1^) and xylazine (10 mg kg^−1^). The eyes were topically anaesthetized with proparacaine and were gently proptosed with forceps. Using a corneal blade and a stereoscope, intrastromal linear keratotomy was performed about 2 mm from the limbus. Using a von Graefe knife (Miltex), a pocket was extended towards the limbus, and the pellet was manoeuvered into the pocket. The wound was coated with a veterinary ophthalmic ointment (Akorn) to prevent infection. Mouse corneas were harvested 7 days after pellet implantation, fixed in 4% paraformaldehyde/PBS (1 h at 4 °C), washed with PBS, and fixed again in iced acetone (15 min at −20 °C). To quantitate the extent of lymphangiogenesis in *Prox1*-EGFP reporter mice, flat mounts of the dissected corneas were evaluated by fluorescence microscopy. Fluorescent images were acquired by EVOS FL cell imaging system (Invitrogen), and vessel areas were calculated using the formula^67^: vessel area=pellet distance × vessel length × clock hours × 0.2π. To quantitate the extent of lymphangiogenesis in WT and PDPN-deficient mice, corneas were stained with eFluor 570-anti-mouse LYVE-1 (clone ALY7, eBioScience, and 1:75) in 10% goat serum/0.2% Triton X-100/PBS overnight, 4 °C. After several washes with 0.2% Triton X-100/PBS, the corneas were flattened and mounted with VECTASHIELD mounting medium (Vector Laboratories) and evaluated by fluorescence microscopy. In some experiments, for comparison purposes, corneas were stained with Alexa Fluor 488-anti-mouse CD31 (clone MEC13.3, 1:100, BioLegend) to visualize blood vessels.

### LEC sprouting assay

LEC spheroids were generated by seeding primary LECs at passage 4 or 5 in each well of 384-well hanging-drop plates (3D Biomatrix) in complete EGM-2 MV medium containing 0.25% methyl cellulose according to the manufacturer's instructions (750 cells per well). After 18 h, LEC spheroids were collected, resuspended in serum-free EBM-2 basal medium, and mixed with collagen solution (PureCol collagen, Advanced BioMatrix, 2.2 mg ml^−1^ in M199 medium, pH adjusted to 7.4 with NaHCO_3_ and NaOH). Aliquots of the LEC spheroid per collagen mixture (250 μl per well of 48-well plate) were incubated in the presence or absence of various test agents for 24 h. At the end of the incubation period, spheroids were stained with Calcein AM (Invitrogen, 1 μg ml^−1^, 37 °C, and 30 min). Fluorescent images were acquired using the EVOS FL cell imaging system, pixels were inverted for better visualization and cumulative length of each sprout was calculated. Results were expressed as fold change compared with corresponding controls. Of note, sprout lengths vary from passages to passages. Test agents used included thiodigalactoside (10–50 mM, Carbosynth), 3′-sialyl lactose (0.2–10 mM, Carbosynth), 6′-sialyl lactose (2–10 mM, Carbosynth), LY294002 (20 μM, Abcam), U0126 (20 μM, Abcam), PD0325901 (1 μM, Selleckchem), anti-integrin α9 functional grade antibody (clone Y9A2, 20 μg ml^−1^, Biolegend), anti-integrin αvβ3 functional grade antibody (clone 23C6, 20 μg ml^−1^, eBioscience), anti-integrin α5 (clone NKI-SAM-1, 20 μg ml^−1^, eBioScience), anti-integrin β5 functional grade antibody (clone KN52, 20 μg ml^−1^, eBioScience), mouse IgG isotype control functional grade antibody (20 μg ml^−1^, eBioscience), Obtustatin (2 μM, R&D Systems), and BIO1211 (10 μM, R&D Systems).

### Corneal transplantation

The mouse model of corneal transplantation was used as previously described^68^. In brief, male BALB/c mice (10-week old) were used as graft recipients and male C57BL/6 mice (10-week old) were used as donors. The donor corneal grafts (2.0 mm) were sutured into 1.5 mm diameter corneal host beds of BALB/c mice recipients with 8–10 interrupted 11-0 nylon sutures. At the conclusion of surgery, the viscoelastic material was exchanged for PBS. Neomycin and polymyxin B sulfates and bacitracin Zinc Ophthalmic Ointment USP (Akorn) were topically applied, and a single eyelid suture (8-0 nylon, Surgical Specialties) was performed. Both eyelid and cornea sutures were removed 1 week after surgery. The mice were treated with PBS or galectin-8 (10 μg for subconjunctival injection and 50 μg for intraperitoneal injection) twice a week beginning postoperative day 7. Grafts were evaluated for signs of rejection by slit-lamp biomicroscopy (SL-D7, Topcon, Tokyo, Japan) once per week. A previously reported scoring system^68^,^69^ was used to grade the degree of corneal clarity, ranging from 0 to 5+ (0, clear graft; 1+, minimal superficial opacity; 2+, mild stromal opacity with pupil margin and iris vessels visible; 3+, moderate stromal opacity with only pupil visible; 4+, intense stromal opacity with the anterior chamber visible; 5+, severe stromal opacity with total obscuration of the anterior chamber). Grafts with an opacity score of 3+ or greater on postoperative week 4 were considered rejected; grafts with opacity scores of 3+ or greater on postoperative week 2 which never cleared were also considered rejected. Mice were killed on postoperative week 4 and corneas were excised for whole-mount staining to visualize and quantify lymphatic and blood vessels.

### Corneal HSV-1 infection

Male WT C57BL/6 and galectin-8 KO mice (10–12-week old) were anaesthetized by intraperitoneal injection of ketamine/Xylazine. Proparacaine eye drop was applied to the corneas as a topical anaesthetic. The corneas were scarified in a grid pattern with a 30-gauge needle, and 3 μl drop containing HSV-1 McKrae strain (1 × 10^6^ pfu, provided by Dr Homayon Ghiasi) was applied to the corneas. The keratitis signs were examined by slit-lamp biomicroscopy on different days post infection (pi). A scoring system ranging from 0 to 4+ was used to grade the degree of corneal clarity (0, normal cornea; 1+, partial corneal opacity covering the pupil; 2+, dense corneal opacity covering the pupil; 3+, dense corneal opacity covering the entire anterior segment; 4+, perforation of the cornea). On day 8 pi, mice were killed and the corneas were excised for whole-mount staining to visualize lymphatic vessels.

### Mouse model of suture-induced inflammatory lymphangiogenesis

The mouse model of suture-induced lymphangiogenesis was used as previously described^70^. In brief, two 11-0 sutures were placed intrastromally about 2 mm from the limbus at the 12 and 6 o'clock positions in the *Prox1*-EGFP reporter mice (8–10-week old). After surgery, a veterinary ophthalmic ointment was applied to prevent infection. Sutures were left in place for 7 days. To assess the effect of galectin inhibitors on suture-induced lymphangiogenesis, 10 μl of vehicle (PBS), TDG (200 mM in PBS), or Gal-8N (15 μg in PBS) were subconjunctivally injected on post-surgery days 0, 2, 4 and 6 using a 32-gauge needle with a 10 μl syringe (Hamilton). On day 7 post surgery, mouse corneas were harvested and processed for staining with anti-LYVE-1 as described above. The areas of lymphatic vessels covering the whole corneas were calculated.

### Affinity precipitation assay

Five milligram of plant lectins (Vector Labs) and galectins were conjugated to 330 mg of Pierce NHS-activated agarose dry resin in accordance with the manufacturer's instructions (Thermo Scientific). Primary LECs (Lonza) were lysed in Triton lysis buffer (20 mM Tris-HCl, pH 7.4; 150 mM NaCl; 0.5% Triton X-100 with protease inhibitor cocktail). LEC lysates (250 μg in 500 μl lysis buffer supplemented with 10 mM MgCl_2_) were incubated with 40 μl (50% slurry) of agarose-conjugated lectins (4 °C, overnight). Beads were pelleted by centrifugation at 3,000 r.p.m. for 1 min. Non-specific binding proteins were removed by washing the beads with Triton lysis buffer once and PBS twice. Supernatants were discarded; bound proteins were eluted by boiling the beads with 20 μl of 2 × Laemmli sample buffer for 7 min, and examined by western blotting.

### Western blot analysis

Primary LECs were lysed with Triton lysis buffer supplemented with protease inhibitor cocktail and Phos-STOP phosphatase inhibitor cocktail (Roche), and subjected to electrophoresis in 4-15% SDS–polyacrylamide gels (Bio-Rad). Protein blots of the gels were blocked with 0.5 × Odyssey blocking buffer (OBB, Li-COR). For AKT and ERK signalling, blots were incubated with primary antibodies (rabbit anti-ERK1/2, 1:7,500, Cell Signaling Technology; mouse anti-phospho-ERK1/2, Thr202/Tyr204, 1:2,000, Cell Signaling Technology; mouse anti-AKT1/2/3, 1:2,000, Cell Signaling Technology; rabbit anti-phospho-AKT, Ser473, 1:1,500, Cell Signaling Technology) overnight at 4 °C. After washing with 0.5% Tween-20/PBS three times, the blots were incubated with appropriate secondary antibodies (donkey anti-rabbit IRDye 680LT and anti-mouse IRDye 800CW, Li-Cor) for 45 min at 25 °C. The blots were scanned by an Odyssey Infrared Imaging System using Image Studio v2.0 software (Li-COR). For integrin signalling, blots were incubated with primary antibodies (rabbit anti-phospho-integrin β1, Tyr783, 1:750, Abcam; rabbit anti-phospho-integrin β1, Thr788/789, 1:750, Invitrogen; goat anti-integrin β1, N-20, 1:1,000, Santa Cruz Biotechnology; mouse anti-FAK, clone 77, 1:2,000, BD Bioscience; anti-phospho-FAK, Tyr397, 1:2,000, Invitrogen) overnight at 4 °C. After washing with 0.5% Tween-20/PBS three times, the blots were incubated with appropriate secondary antibodies for 45 min at 25 °C. The blots were scanned by an Odyssey Infrared Imaging System using Image Studio v2.0 software (Li-COR).

After scanning, the blots were stripped with NewBlot Nitrocellulose stripping buffer (25 °C, 10 min, Li-Cor) and were reprobed with primary antibodies (rabbit anti-VEGFR-3, clone C-20, 1:500, Santa Cruz Biotechnology; rat anti-human PDPN, 1:2,000, BioLegend; mouse anti-GAPDH, clone 6C5, 1:10,000, Santa Cruz Biotechnology; mouse anti-β-actin, clone AC-15, 1:10,000, Santa Cruz Biotechnology) overnight at 4 °C. The blots were developed using appropriate secondary antibodies (goat anti-rabbit IRDye 800CW; anti-rat IRDye 800CW; anti-mouse IRDye 680LT, 1:10,000, Li-Cor) for 45 min at 25 °C. Signals were detected by Odyssey Infrared Imaging System.

### LEC migration assay

Transwell (6.5 mm) with 8 μm-pore polycarbonate membrane inserts (Corning) were used in the migration assay. The lower side of the insert membranes were coated with 400 μl of fibronectin (10 μg ml^−1^, Sigma) or galectin-8 (0.5 μM) (37 °C, overnight), and then the inserts were blocked with 0.1% BSA in PBS, 37 °C, 3 h. LECs were serum-starved in serum-free EBM-2 medium overnight, detached with StemPro Accutase cell dissociation reagent, and resuspended in serum-free EBM-2 medium (2 × 10^5^ cells per ml). Aliquots of LEC suspension (200 μL of 2 × 10^5^ cells per ml) were added to the upper chamber. The bottom chamber was filled with 600 μl of serum-free EBM-2 and the plates were incubated at 37 °C for 2 h. Inserts were fixed in absolute methanol (6 min, 25 °C) and stained with Giemsa stain (40 min, 25 °C, Sigma) per manufacturer's instructions. Membranes were wiped free of cells on the upper surface and mounted with Permount mounting medium (Fisher) on glass slides. The number of migrating cells in each condition was counted in 4 random fields at 10 × magnification, averaged, and normalized to control condition to generate percent-change in migration activity. In some experiments, the cells were incubated in the presence of isotype control Ab (10 μg ml^−1^, eBioScience) or the anti-PDPN functional blocking Ab (10 μg ml^−1^, eBioScience). No LECs attached to BSA-coated membranes, and therefore this group was not included in the graphs.

### Immunoprecipitation

Mouse isotype antibody (15 μg, Santa Cruz Biotechnology) and anti-PDPN (15 μg, clone E-1, Santa Cruz Biotechnology) were immobilized onto AminoLink Plus coupling resin (Pierce) by sodium cyanoborohydride according to manufacturer's instructions. Primary LECs were serum-starved overnight and treated with galectin-8 (0.2 μM) for 15 min at 37 °C. After treatment, cells were lysed with IP lysis/wash buffer (25 mM Tris, 150 mM NaCl, 1 mM EDTA, 1% NP-40, 5% glycerol; pH 7.4) supplemented with protease inhibitor cocktails (Roche). After centrifugation (10 min, 13,000 r.p.m.), supernatants (500 μg protein lysates) were pre-cleared by incubation for 1 h with Pierce control agarose resin at 4 °C. The clarified samples were incubated with the Ab-conjugated agarose resins overnight at 4 °C. Immunoprecipitates were washed three times with IP lysis/wash buffer and once with conditioning buffer provided by the kit (Pierce). The bound proteins were eluted with low pH elution buffer, neutralized with Tris-HCl (pH 9.0), and analysed alongside with inputs by Western blotting using anti-integrin α1 (clone 639508, 1:500, R&D Systems), anti-integrin α5 (clone H-104, 1:1,000, Santa Cruz Biotechnology), anti-integrin β1 (clone N-20, 1:1,000, Santa Cruz Biotechnology), anti-integrin α9 (ab87995, 1:500, Abcam), anti-integrin β3 (clone H-96, 1:1,000, Santa Cruz Biotechnology), anti-integrin αv (clone 21, 1:1,000, BD Biosciences), anti-PDPN, anti-galectin-8 (clone NBP1-66520, 1:1,000, Novus Biologicals) and anti-GAPDH as describe above.

### Immunocytochemistry staining

Primary LECs were treated with galectin-8 (0.5 μM) for 15 min at 37 °C, washed with PBS three times, and fixed with 4% paraformaldehyde/PBS (10 min, 25 °C). Cells were blocked with Image-iT FX signal enhancer (30 min, 25 °C, Invitrogen) and incubated with primary antibodies (rat anti-human PDPN, 1:300, BioLegend; mouse anti-human VEGFR-3, 1:100, clone 9D9F9, BioLegend; mouse anti-integrin β1, 1:100, clone 18/CD29, BD Biosciences; rabbit anti-galectin-8, H-80, 1:100, Santa Cruz Biotechnology) in 2% BSA/PBS at 4 °C overnight. After this, the cells were washed with 2% BSA/PBS three times and incubated with appropriate secondary antibodies (Alexa Fluor 488-anti-mouse, 1:200, Invitrogen; Alexa Fluor® 568 anti-rabbit, 1:200, Invitrogen; Alexa Fluor 647 anti-rat, 1:300, Jackson ImmunoResearch) at 25 °C for 1 h. After washing with PBS, slides were mounted with ProLong Gold antifade reagent with DAPI (Invitrogen) and examined with the Leica TCS SPE imaging system (Leica).

### Flow cytometry analysis

To determine if PDPN deficiency alters cell surface expression of integrins and VEGFR-3, flow cytometry analysis was used. Primary LECs were transfected with control siRNA or PDPN siRNA as described above. After 48 h transfection, the cells were lifted with StemPro Accutase cell dissociation reagent (Invitrogen), washed with PBS and fixed with 2% paraformaldehyde/PBS on ice for 10 min, and were then stained using rat anti-human PDPN (1:500, BioLegend), mouse anti-human VEGFR-3 (clone 9D9F9, 1:100, BioLegend), mouse anti-human integrin α5 (clone NKI-SAM-1, 1:300, BioLegend) and mouse anti-human integrin β1 (clone TS2/16, 1:300, BioLegend) primary antibodies in cell staining buffer (BioLegend, 45 min on ice) and Alexa Fluor 488 donkey anti-rat and Alexa Fluor 647 anti-mouse secondary antibodies (1:1,000, Invitrogen, 30 min on ice). For negative controls, isotype antibodies were used. The stained cells were fixed with 2% paraformaldehyde/PBS, analysed with BD FACSCalibur, and the mean fluorescence intensity of VEGFR-3 and PDPN were quantified with the FlowJo software (version 9.5.2).

### Statistics

Data in all figures are presented as mean±s.e.m.. All results were confirmed in 2 or more independent experiments. Data were analysed using paired two-tailed Student's *t*-test or one-way analysis of variance in Prism 6 (GraphPad) as indicated in figure legends. *P* value <0.05 was considered statistically significant.

## Additional information

**Accession codes:** RNA-seq data have been deposited in the Gene Expression Omnibus (GEO) database under accession code GSE79160.

**How to cite this article:** Chen, W.-S. *et al*. Pathological lymphangiogenesis is modulated by galectin-8-dependent crosstalk between podoplanin and integrin-associated VEGFR-3. *Nat. Commun.* 7:11302 doi: 10.1038/ncomms11302 (2016).

## Supplementary Material

Supplementary Information Supplementary Figures 1-11, Supplementary Methods and Supplementary References

Supplementary Data 1 RNA-Seq data comparing transcriptome of control and podoplanin knockdown lymphatic endothelial cells.

## References

[b1] Kerjaschki D. The lymphatic vasculature revisited. J. Clin. Invest. 124, 874–877 (2014). 24590271 10.1172/JCI74854 PMC3938252

[b2] Aspelund A. . The Schlemm's canal is a VEGF-C/VEGFR-3-responsive lymphatic-like vessel. J. Clin. Invest. 124, 3975–3986 (2014). 25061878 10.1172/JCI75395 PMC4153703

[b3] Dietrich T. . Cutting edge: lymphatic vessels, not blood vessels, primarily mediate immune rejections after transplantation. J. Immunol. 184, 535–539 (2010). 20018627 10.4049/jimmunol.0903180 PMC4725297

[b4] Goyal S., Chauhan S. K. & Dana R. Blockade of prolymphangiogenic vascular endothelial growth factor C in dry eye disease. Arch. Ophthalmol. 130, 84–89 (2012). 21911653 10.1001/archophthalmol.2011.266 PMC3629840

[b5] Lee H. S. . Involvement of corneal lymphangiogenesis in a mouse model of allergic eye disease. Invest. Ophthalmol. Vis. Sci. 56, 3140–3148 (2015). 26024097 10.1167/iovs.14-16186 PMC4451613

[b6] Thomson B. R. . A lymphatic defect causes ocular hypertension and glaucoma in mice. J. Clin. Invest. 124, 4320–4324 (2014). 25202984 10.1172/JCI77162 PMC4191022

[b7] Wuest T. R. & Carr D. J. VEGF-A expression by HSV-1-infected cells drives corneal lymphangiogenesis. J. Exp. Med. 207, 101–115 (2010). 20026662 10.1084/jem.20091385 PMC2812544

[b8] Witte M. H. . Lymphangiogenesis and hemangiogenesis: potential targets for therapy. J. Surg. Oncol. 103, 489–500 (2011). 21480241 10.1002/jso.21714 PMC4422163

[b9] Markowska A. I., Jefferies K. C. & Panjwani N. Galectin-3 protein modulates cell surface expression and activation of vascular endothelial growth factor receptor 2 in human endothelial cells. J. Biol. Chem. 286, 29913–29921 (2011). 21715322 10.1074/jbc.M111.226423 PMC3191032

[b10] Markowska A. I., Liu F. T. & Panjwani N. Galectin-3 is an important mediator of VEGF- and bFGF-mediated angiogenic response. J. Exp. Med. 207, 1981–1993 (2010). 20713592 10.1084/jem.20090121 PMC2931172

[b11] Ideo H., Seko A., Ishizuka I. & Yamashita K. The N-terminal carbohydrate recognition domain of galectin-8 recognizes specific glycosphingolipids with high affinity. Glycobiology 13, 713–723 (2003). 12851289 10.1093/glycob/cwg094

[b12] Ideo H., Matsuzaka T., Nonaka T., Seko A. & Yamashita K. Galectin-8-N-domain recognition mechanism for sialylated and sulfated glycans. J. Biol. Chem. 286, 11346–11355 (2011). 21288902 10.1074/jbc.M110.195925 PMC3064191

[b13] Carlsson S. . Affinity of galectin-8 and its carbohydrate recognition domains for ligands in solution and at the cell surface. Glycobiology 17, 663–676 (2007). 17339281 10.1093/glycob/cwm026

[b14] Cueni L. N. & Detmar M. Galectin-8 interacts with podoplanin and modulates lymphatic endothelial cell functions. Exp. Cell Res. 315, 1715–1723 (2009). 19268462 10.1016/j.yexcr.2009.02.021 PMC3398156

[b15] Cueni L. N. . Podoplanin-Fc reduces lymphatic vessel formation *in vitro* and *in vivo* and causes disseminated intravascular coagulation when transgenically expressed in the skin. Blood 116, 4376–4384 (2010). 20716773 10.1182/blood-2010-04-278564 PMC2993634

[b16] Breiteneder-Geleff S. . Podoplanin, novel 43-kd membrane protein of glomerular epithelial cells, is down-regulated in puromycin nephrosis. Am. J. Pathol. 151, 1141–1152 (1997). 9327748 PMC1858024

[b17] Schacht V. . T1alpha/podoplanin deficiency disrupts normal lymphatic vasculature formation and causes lymphedema. EMBO J. 22, 3546–3556 (2003). 12853470 10.1093/emboj/cdg342 PMC165612

[b18] Fu J. . Endothelial cell O-glycan deficiency causes blood/lymphatic misconnections and consequent fatty liver disease in mice. J. Clin. Invest. 118, 3725–3737 (2008). 18924607 10.1172/JCI36077 PMC2567837

[b19] Navarro A., Perez R. E., Rezaiekhaligh M., Mabry S. M. & Ekekezie I. I. T1alpha/podoplanin is essential for capillary morphogenesis in lymphatic endothelial cells. Am. J. Physiol. Lung Cell Mol. Physiol. 295, L543–L551 (2008). 18658274 10.1152/ajplung.90262.2008

[b20] Navarro A., Perez R. E., Rezaiekhaligh M. H., Mabry S. M. & Ekekezie I. I. Polarized migration of lymphatic endothelial cells is critically dependent on podoplanin regulation of Cdc42. Am. J. Physiol. Lung Cell Mol. Physiol. 300, L32–L42 (2011). 21036919 10.1152/ajplung.00171.2010

[b21] Maruyama Y. . The effect of podoplanin inhibition on lymphangiogenesis under pathological conditions. Invest. Ophthalmol. Vis. Sci. 55, 4813–4822 (2014). 24985477 10.1167/iovs.13-13711

[b22] Kaneko M. K. . Functional glycosylation of human podoplanin: glycan structure of platelet aggregation-inducing factor. FEBS Lett. 581, 331–336 (2007). 17222411 10.1016/j.febslet.2006.12.044

[b23] Pan Y. . Podoplanin requires sialylated O-glycans for stable expression on lymphatic endothelial cells and for interaction with platelets. Blood 124, 3656–3665 (2014). 25336627 10.1182/blood-2014-04-572107 PMC4256915

[b24] Chen J., Alexander J. S. & Orr A. W. Integrins and their extracellular matrix ligands in lymphangiogenesis and lymph node metastasis. Int. J. Cell Biol. 2012, 853703 (2012). 22505936 10.1155/2012/853703 PMC3296286

[b25] Wang J. F., Zhang X. F. & Groopman J. E. Stimulation of beta 1 integrin induces tyrosine phosphorylation of vascular endothelial growth factor receptor-3 and modulates cell migration. J. Biol. Chem. 276, 41950–41957 (2001). 11553610 10.1074/jbc.M101370200

[b26] Zhang X., Groopman J. E. & Wang J. F. Extracellular matrix regulates endothelial functions through interaction of VEGFR-3 and integrin alpha5beta1. J. Cell. Physiol. 202, 205–214 (2005). 15389531 10.1002/jcp.20106

[b27] Troncoso M. F. . Galectin-8: a matricellular lectin with key roles in angiogenesis. Glycobiology 24, 907–914 (2014). 24939370 10.1093/glycob/cwu054

[b28] Kim I. . Molecular cloning, expression, and characterization of angiopoietin-related protein. angiopoietin-related protein induces endothelial cell sprouting. J. Biol. Chem. 274, 26523–26528 (1999). 10473614 10.1074/jbc.274.37.26523

[b29] Abdel-Malak N. A. . Angiopoietin-1 promotes endothelial cell proliferation and migration through AP-1-dependent autocrine production of interleukin-8. Blood 111, 4145–4154 (2008). 18252863 10.1182/blood-2007-08-110338

[b30] Yoon C. M. . Sphingosine-1-phosphate promotes lymphangiogenesis by stimulating S1P1/Gi/PLC/Ca2+ signaling pathways. Blood 112, 1129–1138 (2008). 18541717 10.1182/blood-2007-11-125203 PMC2515114

[b31] Carlsson S., Carlsson M. C. & Leffler H. Intracellular sorting of galectin-8 based on carbohydrate fine specificity. Glycobiology 17, 906–912 (2007). 17580315 10.1093/glycob/cwm059

[b32] Partridge E. A. . Regulation of cytokine receptors by Golgi N-glycan processing and endocytosis. Science 306, 120–124 (2004). 15459394 10.1126/science.1102109

[b33] Yang R. Y., Hill P. N., Hsu D. K. & Liu F. T. Role of the carboxyl-terminal lectin domain in self-association of galectin-3. Biochemistry 37, 4086–4092 (1998). 9521730 10.1021/bi971409c

[b34] Levy Y. . It depends on the hinge: a structure-functional analysis of galectin-8, a tandem-repeat type lectin. Glycobiology 16, 463–476 (2006). 16501058 10.1093/glycob/cwj097

[b35] Deng Y., Atri D., Eichmann A. & Simons M. Endothelial ERK signaling controls lymphatic fate specification. J. Clin. Invest. 123, 1202–1215 (2013). 23391722 10.1172/JCI63034 PMC3582116

[b36] Zhou F. . Akt/protein kinase B is required for lymphatic network formation, remodeling, and valve development. Am. J. Pathol. 177, 2124–2133 (2010). 20724596 10.2353/ajpath.2010.091301 PMC2947305

[b37] Singh N. . Soluble vascular endothelial growth factor receptor 3 is essential for corneal alymphaticity. Blood 121, 4242–4249 (2013). 23476047 10.1182/blood-2012-08-453043 PMC3656456

[b38] Hajrasouliha A. R. . Vascular endothelial growth factor-C promotes alloimmunity by amplifying antigen-presenting cell maturation and lymphangiogenesis. Invest. Ophthalmol. Vis. Sci. 53, 1244–1250 (2012). 22281820 10.1167/iovs.11-8668 PMC3339906

[b39] Liesegang T. J., Melton L. J. 3rd, Daly P. J. & Ilstrup D. M. Epidemiology of ocular herpes simplex. Incidence in Rochester, Minn, 1950 through 1982. Arch. Ophthalmol. 107, 1155–1159 (1989). 2787981 10.1001/archopht.1989.01070020221029

[b40] Park P. J. . Corneal lymphangiogenesis in herpetic stromal keratitis. Surv. Ophthalmol. 60, 60–71 (2015). 25444520 10.1016/j.survophthal.2014.06.001 PMC4262646

[b41] Cursiefen C. . VEGF-A stimulates lymphangiogenesis and hemangiogenesis in inflammatory neovascularization via macrophage recruitment. J. Clin. Invest. 113, 1040–1050 (2004). 15057311 10.1172/JCI20465 PMC379325

[b42] Albuquerque R. J. . Alternatively spliced vascular endothelial growth factor receptor-2 is an essential endogenous inhibitor of lymphatic vessel growth. Nat. Med. 15, 1023–1030 (2009). 19668192 10.1038/nm.2018 PMC2882165

[b43] Garner O. B. & Baum L. G. Galectin-glycan lattices regulate cell-surface glycoprotein organization and signalling. Biochem. Soc. Trans. 36, 1472–1477 (2008). 19021578 10.1042/BST0361472 PMC2811491

[b44] Rabinovich G. A., Toscano M. A., Jackson S. S. & Vasta G. R. Functions of cell surface galectin-glycoprotein lattices. Curr. Opin. Struct. Biol. 17, 513–520 (2007). 17950594 10.1016/j.sbi.2007.09.002 PMC2100406

[b45] Joukov V. . Proteolytic processing regulates receptor specificity and activity of VEGF-C. EMBO J. 16, 3898–3911 (1997). 9233800 10.1093/emboj/16.13.3898 PMC1170014

[b46] Wirzenius M. . Distinct vascular endothelial growth factor signals for lymphatic vessel enlargement and sprouting. J. Exp. Med. 204, 1431–1440 (2007). 17535974 10.1084/jem.20062642 PMC2118625

[b47] Vlahakis N. E., Young B. A., Atakilit A. & Sheppard D. The lymphangiogenic vascular endothelial growth factors VEGF-C and -D are ligands for the integrin alpha9beta1. J. Biol. Chem. 280, 4544–4552 (2005). 15590642 10.1074/jbc.M412816200 PMC1368959

[b48] Karpanen T. . Functional interaction of VEGF-C and VEGF-D with neuropilin receptors. FASEB J. 20, 1462–1472 (2006). 16816121 10.1096/fj.05-5646com

[b49] Herzog B. H. . Podoplanin maintains high endothelial venule integrity by interacting with platelet CLEC-2. Nature 502, 105–109 (2013). 23995678 10.1038/nature12501 PMC3791160

[b50] Reynolds A. R. Potential relevance of bell-shaped and u-shaped dose-responses for the therapeutic targeting of angiogenesis in cancer. Dose Response 8, 253–284 (2009). 20877487 10.2203/dose-response.09-049.Reynolds PMC2939687

[b51] Hirabayashi J. . Oligosaccharide specificity of galectins: a search by frontal affinity chromatography. Biochim. Biophys. Acta 1572, 232–254 (2002). 12223272 10.1016/s0304-4165(02)00311-2

[b52] Brewer C. F., Miceli M. C. & Baum L. G. Clusters, bundles, arrays and lattices: novel mechanisms for lectin-saccharide-mediated cellular interactions. Curr. Opin. Struct. Biol. 12, 616–623 (2002). 12464313 10.1016/s0959-440x(02)00364-0

[b53] Yamagami S. & Dana M. R. The critical role of lymph nodes in corneal alloimmunization and graft rejection. Invest. Ophthalmol. Vis. Sci. 42, 1293–1298 (2001). 11328742

[b54] Yamagami S., Dana M. R. & Tsuru T. Draining lymph nodes play an essential role in alloimmunity generated in response to high-risk corneal transplantation. Cornea 21, 405–409 (2002). 11973391 10.1097/00003226-200205000-00014

[b55] Emami-Naeini P. . Soluble vascular endothelial growth factor receptor-3 suppresses allosensitization and promotes corneal allograft survival. Graefes Arch. Clin. Exp. Ophthalmol. 252, 1755–1762 (2014). 25091513 10.1007/s00417-014-2749-5 PMC4221529

[b56] Bryant-Hudson K. M., Gurung H. R., Zheng M. & Carr D. J. Tumor necrosis factor alpha and interleukin-6 facilitate corneal lymphangiogenesis in response to herpes simplex virus 1 infection. J. Virol. 88, 14451–14457 (2014). 25297992 10.1128/JVI.01841-14 PMC4249127

[b57] Ling S. . Development of new lymphatic vessels in alkali-burned corneas. Acta Ophthalmol. 87, 315–322 (2009). 18811642 10.1111/j.1755-3768.2008.01349.x

[b58] Kerjaschki D. . Lymphatic neoangiogenesis in human kidney transplants is associated with immunologically active lymphocytic infiltrates. J. Am. Soc. Nephrol. 15, 603–612 (2004). 14978162 10.1097/01.asn.0000113316.52371.2e

[b59] Kerjaschki D. . Lymphatic endothelial progenitor cells contribute to de novo lymphangiogenesis in human renal transplants. Nat. Med. 12, 230–234 (2006). 16415878 10.1038/nm1340

[b60] Nykanen A. I. . Targeting lymphatic vessel activation and CCL21 production by vascular endothelial growth factor receptor-3 inhibition has novel immunomodulatory and antiarteriosclerotic effects in cardiac allografts. Circulation 121, 1413–1422 (2010). 20231530 10.1161/CIRCULATIONAHA.109.910703

[b61] Choi I. . Visualization of lymphatic vessels by Prox1-promoter directed GFP reporter in a bacterial artificial chromosome-based transgenic mouse. Blood 117, 362–365 (2011). 20962325 10.1182/blood-2010-07-298562 PMC3037757

[b62] Valenzuela D. M. . High-throughput engineering of the mouse genome coupled with high-resolution expression analysis. Nat. Biotechnol. 21, 652–659 (2003). 12730667 10.1038/nbt822

[b63] Poland P. A., Kinlough C. L. & Hughey R. P. Cloning, expression, and purification of galectins for in vitro studies. Methods Mol. Biol. 1207, 37–49 (2015). 25253131 10.1007/978-1-4939-1396-1_2

[b64] Paranthan R. R., Bargagna-Mohan P., Lau D. L. & Mohan R. A robust model for simultaneously inducing corneal neovascularization and retinal gliosis in the mouse eye. Mol. Vis. 17, 1901–1908 (2011). 21850164 PMC3144731

[b65] Panjwani N., Moulton P., Alroy J. & Baum J. Localization of lectin binding sites in human, cat, and rabbit corneas. Invest. Ophthalmol. Vis. Sci. 27, 1280–1284 (1986). 2426217

[b66] Cao R. . Mouse corneal lymphangiogenesis model. Nat. Protoc. 6, 817–826 (2011). 21637201 10.1038/nprot.2011.359

[b67] Rogers M. S., Birsner A. E. & D'Amato R. J. The mouse cornea micropocket angiogenesis assay. Nat. Protoc. 2, 2545–2550 (2007). 17947997 10.1038/nprot.2007.368

[b68] Sugaya S. . Comparison of galectin expression signatures in rejected and accepted murine corneal allografts. Cornea 34, 675–681 (2015). 25961492 10.1097/ICO.0000000000000439 PMC4430336

[b69] Sano Y., Ksander B. R. & Streilein J. W. Minor H, rather than MHC, alloantigens offer the greater barrier to successful orthotopic corneal transplantation in mice. Transpl. Immunol. 4, 53–56 (1996). 8762011 10.1016/s0966-3274(96)80035-9

[b70] Cursiefen C. . Roles of thrombospondin-1 and -2 in regulating corneal and iris angiogenesis. Invest. Ophthalmol. Vis. Sci. 45, 1117–1124 (2004). 15037577 10.1167/iovs.03-0940

