# Erratum: Pathological lymphangiogenesis is modulated by galectin-8-dependent crosstalk between podoplanin and integrin-associated VEGFR-3

**DOI:** 10.1038/ncomms12063

**Published:** 2016-07-19

**Authors:** Wei-Sheng Chen, Zhiyi Cao, Satoshi Sugaya, Maria J. Lopez, Victor G. Sendra, Nora Laver, Hakon Leffler, Ulf J. Nilsson, Jianxin Fu, Jianhua Song, Lijun Xia, Pedram Hamrah, Noorjahan Panjwani

Nature Communications 7: Article number: 1130210.1038/ncomms11302 (2016); Published: 04 12 2016; Updated: 07 19 2016.

In Fig. 6a of this Article the sixth histogram bars were accidentally mislabeled during the production process. The label ‘VEGF-C+anti-β3' should read ‘VEGF-C+anti-β5', and ‘Gal-8+anti-β3' should read ‘Gal-8+anti-β5'.

The correct version of the figure appears below.


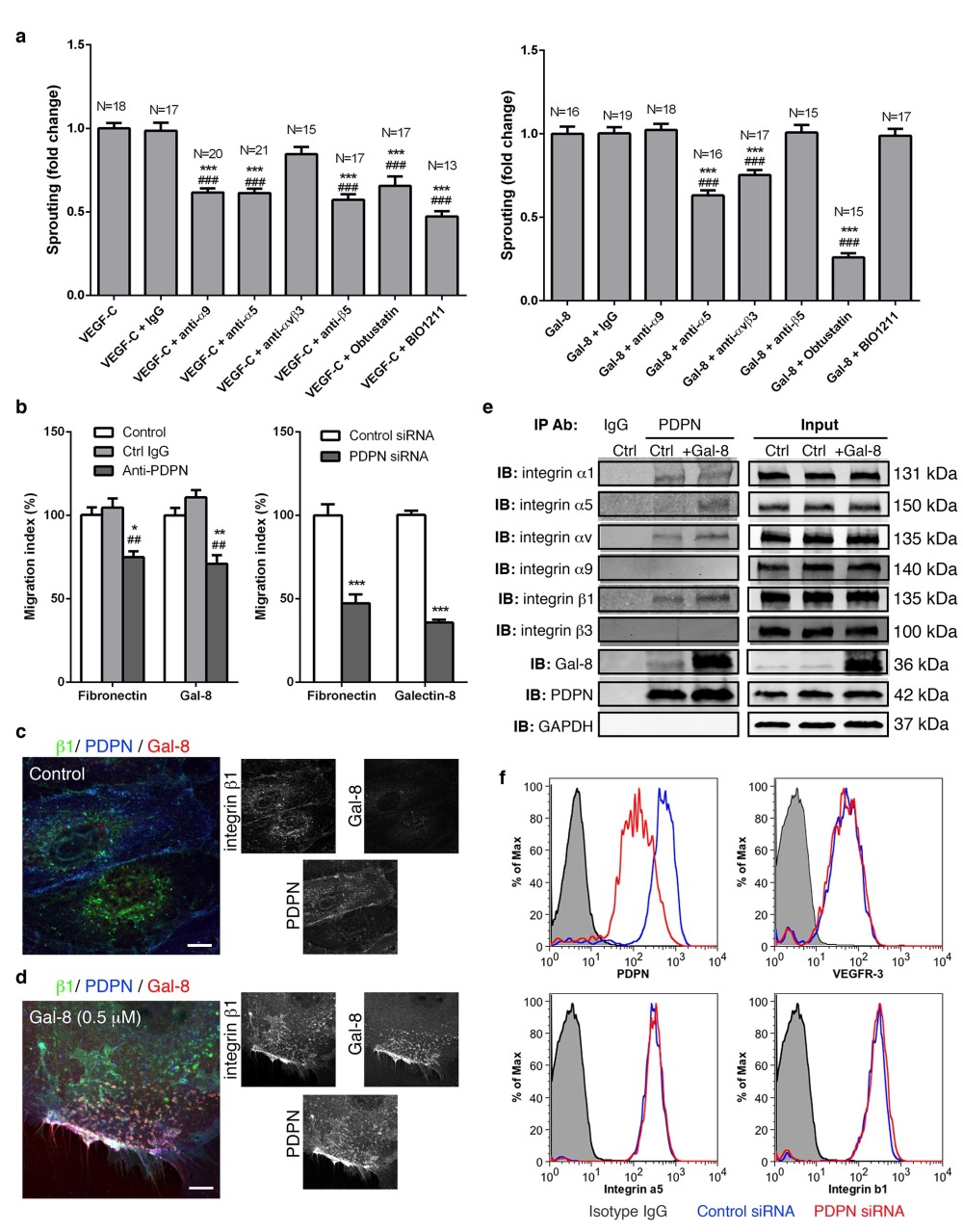


**Figure 6**

