# Supplementary material for: Pathological lymphangiogenesis is modulated by galectin-8-dependent crosstalk between podoplanin and integrin-associated VEGFR-3
Source: Nat Commun. 2016 Apr 12;7:11302. doi: 10.1038/ncomms11302 (PMC4832077; doi:10.1038/ncomms11302)
Supplement: Supplementary Information — Supplementary Figures 1-11, Supplementary Methods and Supplementary References [file ncomms11302-s1.pdf]

Supplementary Information

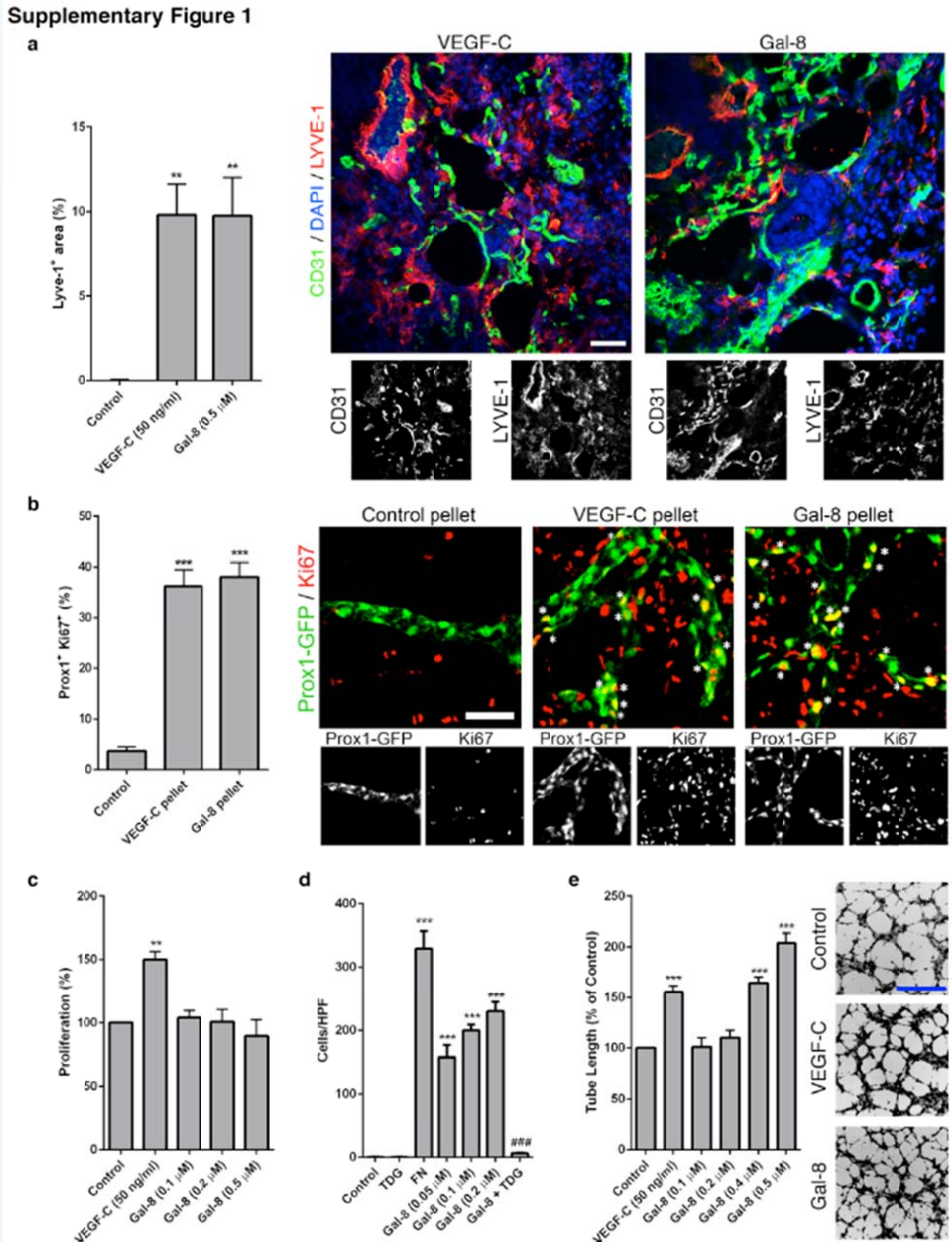

Supplementary figure 1. Galectin-8 promotes lymphangiogenesis in vivo and in vitro. (**a**) Galectin-8 induces lymphangiogenesis in Matrigel plug assay. Matrigel mixed with PBS, VEGF-C (50 ng per ml) and galectin-8 (0.5 μM) was subcutaneously injected into C57BL/6 mice. Frozen sections of the Matrigel plugs on day 7 post-injection were stained with anti-CD31 (green) and anti-LYVE-1 (red), and LYVE-1^{+} areas (indicating lymphatic vessels) were quantified by ImageJ. Representative fluorescence images are shown in the right panels. *N*=4 for each group. (**b**) Galectin-8 promotes LEC proliferation in vivo. Sustained-release polymer pellets containing galectin-8 or VEGF-C were implanted in the corneas of *Prox1*-EGFP reporter mice. One week after surgery, the mouse corneas were stained with anti-Ki67 (red). Proliferative LECs (*Prox1*-$GFP^{+}Ki67^{+}$, yellow, white asterisk) in the lymphangiogenic area induced by VEGF-C or galectin-8 were enumerated (left panel). Quiescent LECs (*Prox1*-$GFP^{+}Ki67^{-}$, green) in the limbus of corneas implanted with control pellets served as a control. Representative fluorescence images of corneas implanted with control, VEGF-C pellet, or galectin-8 pellet are shown in the right panel. *N*=4 or more for each group. (**c**) Galectin-8 does not promote LEC proliferation in vitro. Cell proliferation was determined by WST-1. Results are expressed as % change where control is set as 100%. *N*=4 for each group. (**d**) Galectin-8 promotes LEC migration (haptotaxis). The lower-side of the insert membrane was coated with 1% bovine serum albumin (control), fibronectin (FN, 10 μg per ml) or different concentrations of galectin-8 in the presence or absence of TDG (20 mM). Galectin-8 induced cells migration in a dose-dependent manner. The stimulatory effect of galectin-8 on cell migration was inhibited by TDG. *N*=4 for each group. (**e**) Galectin-8 promotes LEC tube formation. LECs were seeded on Matrigel coated 8-chamber slides in the presence of VEGF-C (50 ng per ml) or varying concentrations of galectin-8 in serum-free EBM-2 medium. Results are expressed as % change where control is set as 100%. *N*=3. Data are plotted as mean ± SEM and analyzed using one-way ANOVA. $^{**}P<0.01$, $^{***}P<0.001$ vs control. $^{\#\#\#}P<0.001$ vs Gal-8 (0.2 μM) (d). Bar: 50 μm (a,b), 1 mm (e).

Supplementary Figure 2

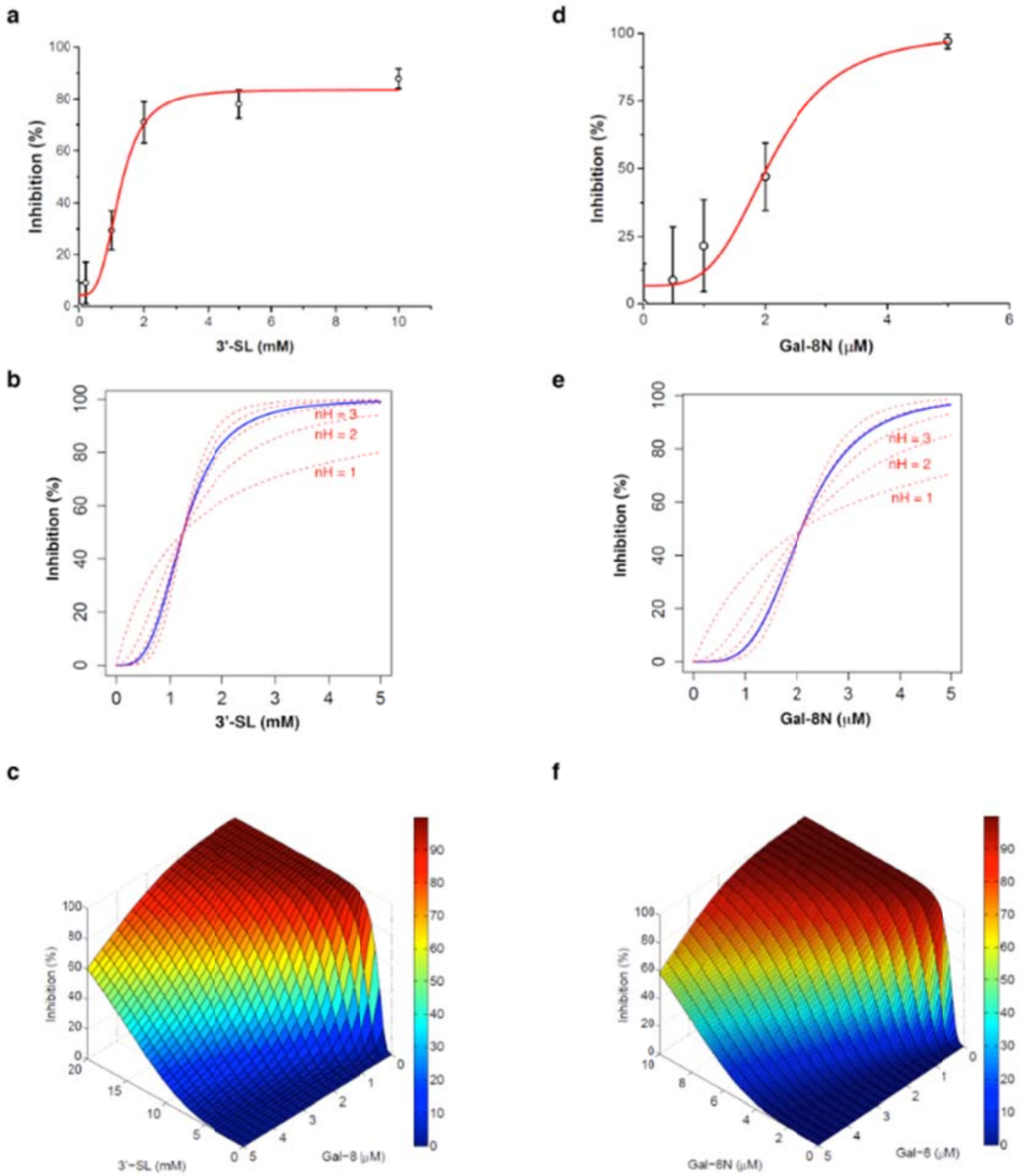

Supplementary figure 2. Kinetic characteristics of inhibitory effect of 3'-SL and Gal-8N on galectin-8-mediated LEC sprouting. Inhibitory effect of 3'-SL (**a**–**c**) and Gal-8N (**d**–**f**) on galectin-8-mediated LEC sprouting at 0.75 μM. Open circles indicate the actual data points. IC_{50} of the inhibitory effect of 3'-SL (a) and Gal-8N (d) are 1.25 mM and 1.98 μM, respectively. nH of 3'-SL and Gal-8N are 3.38 and 3.85, respectively. Ki of 3'-SL and Gal-8N are 1.67 mM and 0.86 μM, respectively. ($Ki = \frac{IC50}{1+[A]/EC50}$). Theoretical curves of different nH (red broken lines, nH=1 to 5) were simulated and the blue lines indicate the inhibitory curves of 3'-SL and Gal-8N based on the experimental results (b,e). Inhibitory effect of 3'-SL (c) and Gal-8N (f) on galectin-8-mediated LEC sprouting at varying concentrations are also simulated. The results are plotted in Origin 9.1 (a,d), R programing language (b,e) and MATLAB2013 (c,f).

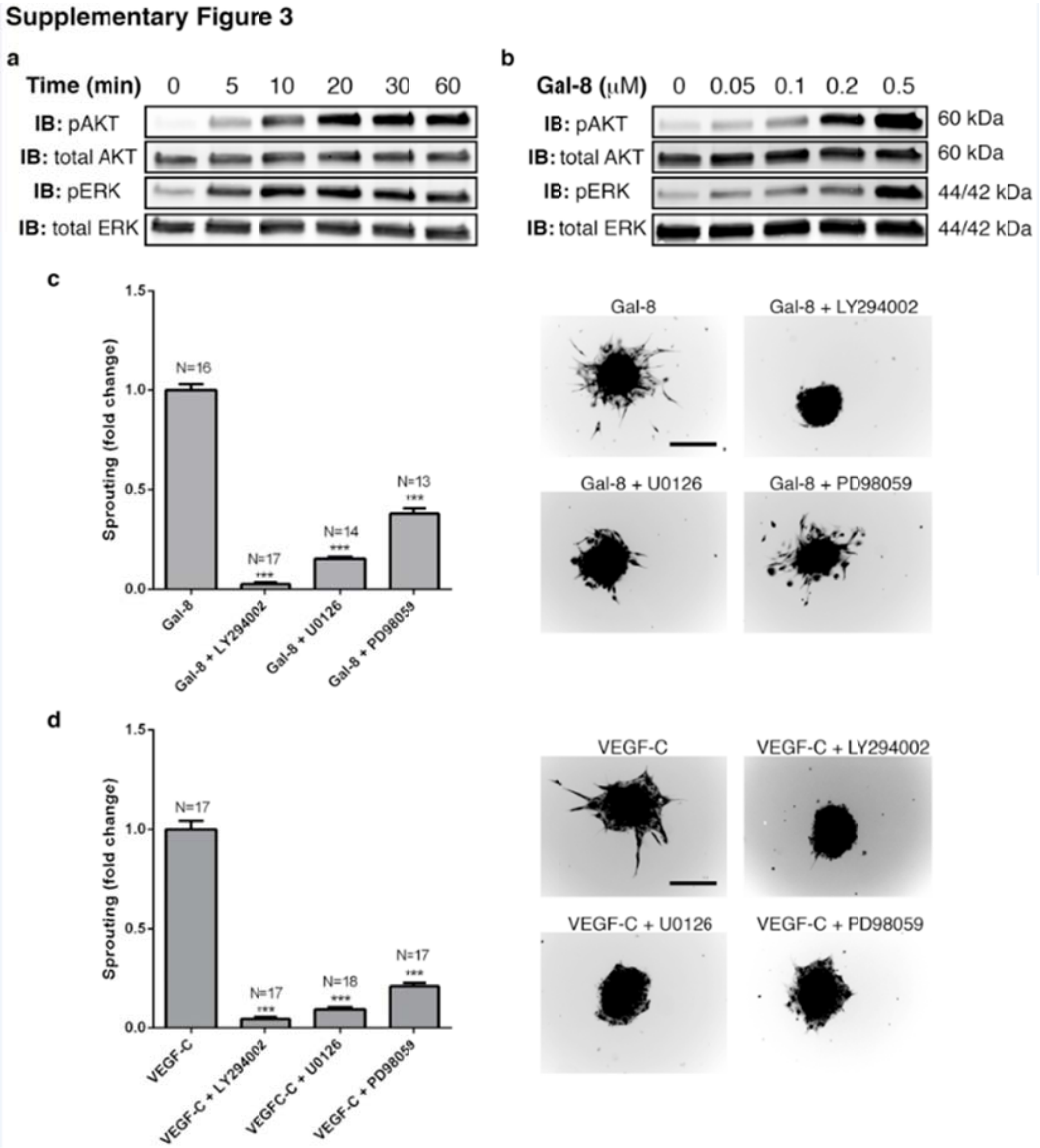

Supplementary figure 3. Galectin-8-induced LEC sprouting is dependent on the activation of AKT and ERK pathways. **(a−b)** Galectin-8 treatment activates AKT and ERK pathways. Primary LECs were treated with galectin-8 prior to lysis. Samples were separated by SDS-PAGE and analyzed by Western blot with phospho-specific antibodies directed against AKT and ERK. (a) LECs were incubated with galectin-8 (0.5 μM) for 0 – 60 minutes prior to lysis. (b) LECs were incubated with varying concentrations of Gal-8 for 30 minutes prior to lysis. **(c)** Inhibitors of PI3K and MEK inhibit galectin-8-induced LEC sprouting. LEC spheroids were stimulated with galectin-8 (0.75 μM) in the presence or absence of PI3K inhibitor (LY294002, 20 μM) and two MEK inhibitors (U0126, 20 μM; PD0325901, 1 μM). A value of 1.0 was assigned to the sprout length of galectin-8 treated cells. The values for inhibitor treated groups are expressed as a change in the sprout length with respect to galectin-8 treated cells. Representative images of sprouts are shown in the right panel. **(d)** Positive control. Same as (c) except that cells were treated with VEGF-C (50 ng per ml). Data are plotted as mean ± SEM and analyzed using one-way ANOVA. ***P<0.001 vs control. The results are representative of two independent experiments. Bar: 100 μm (c,d).

Supplement Figure 4

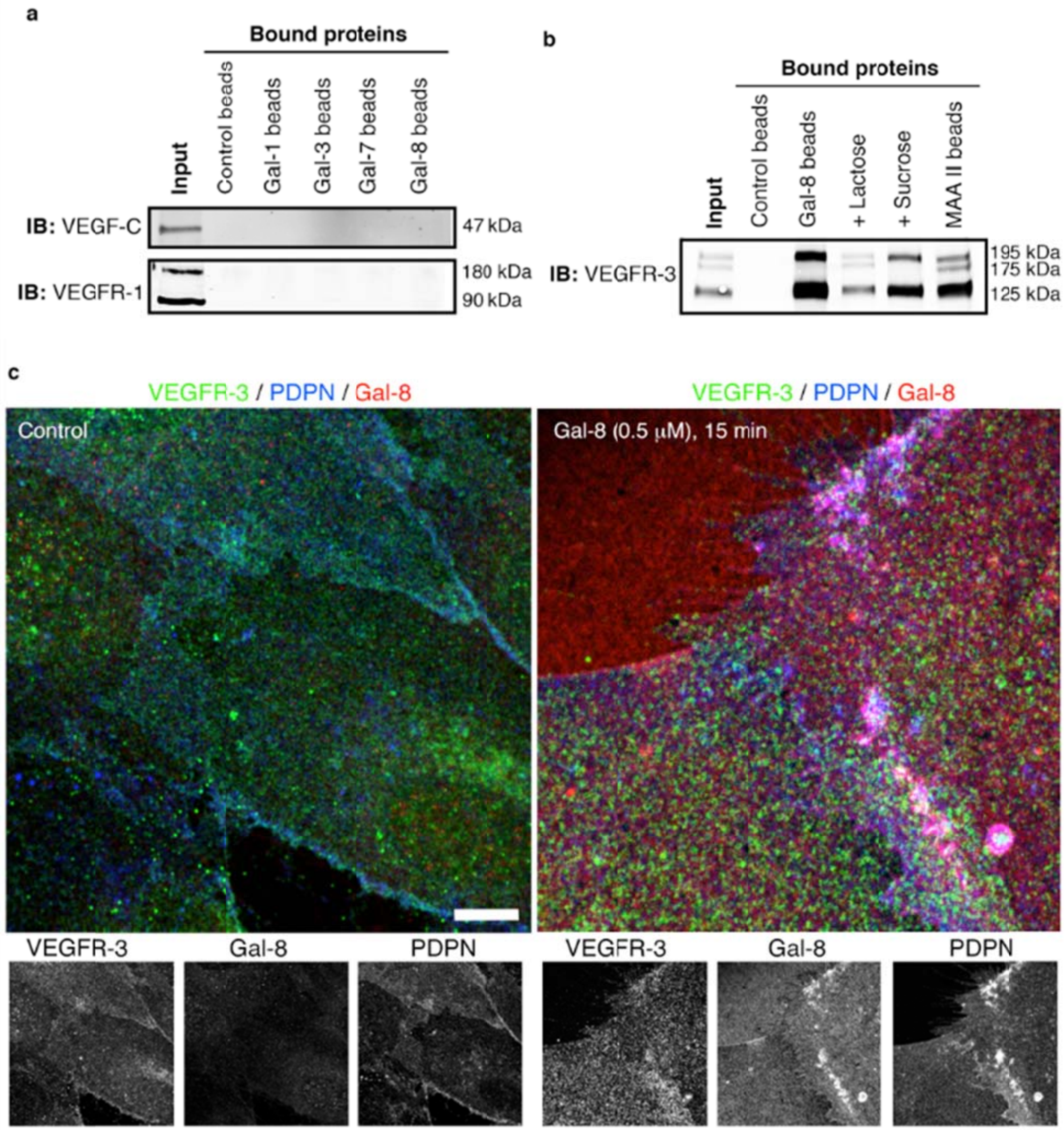

Supplementary figure 4. Galectin-8 interacts with VEGFR-3. (a) None of the four galectins tested bind VEGF-C. Since VEGFR-1 indirectly promotes lymphangiogenesis by recruiting macrophages (Murakami et al., 2008)[1], we also examined whether VEGFR-1 binds to one or more galectins. VEGFR-1 did not interact with the four lectins. Primary LECs were incubated with agarose beads (control), and galectin-conjugated agarose beads at 4^0C overnight. Unbound proteins were removed and the bound proteins were eluted with 20 µl of 2× Laemmli sample buffer and examined along with input by Western blotting. The results are representative of two independent experiments. **(b)** VEGFR-3 is a galectin-8 binding protein. LEC lysates were

incubated with galectin-8-conjugated agarose beads in the presence or absence of 100 mM of lactose (an inhibiting sugar) or sucrose (a non-inhibiting sugar). Bound proteins were examined along with total cell lysates (input) by Western blot using anti-VEGFR-3 antibody. LEC lysates incubated with unconjugated beads served as a negative control. To determine whether VEGFR-3 contains α2,3-sialylated glycans, preferred ligands of galectin-8, binding of VEGFR-3 to a plant lectin, MAA II, which binds selectively to α2,3-linked sialic acids was examined. MAA II, *Maakia Amurensis* agglutinin II. The results are representative of three independent experiments. (**c**) Galectin-8 clusters VEGFR-3 and PDPN on cell surface. LECs were treated with or without galectin-8 for 15 min, fixed without permeabilization, stained with antibodies to anti-VEGFR-3 (green), PDPN (blue) and galectin-8 (red), and examined by confocal microscopy. Merged images are shown in the top panel. In control LECs, no obvious colocalization of VEGFR-3, galectin-8 and PDPN. In contrast, exogenous galectin-8 sequesters VEGFR-3 and PDPN. PDPN and VEGFR-3 distribution reorganized and colocalized (white spots in the merged image) at cell borders after galectin-8 treatment. Bar: 7.5 µm.

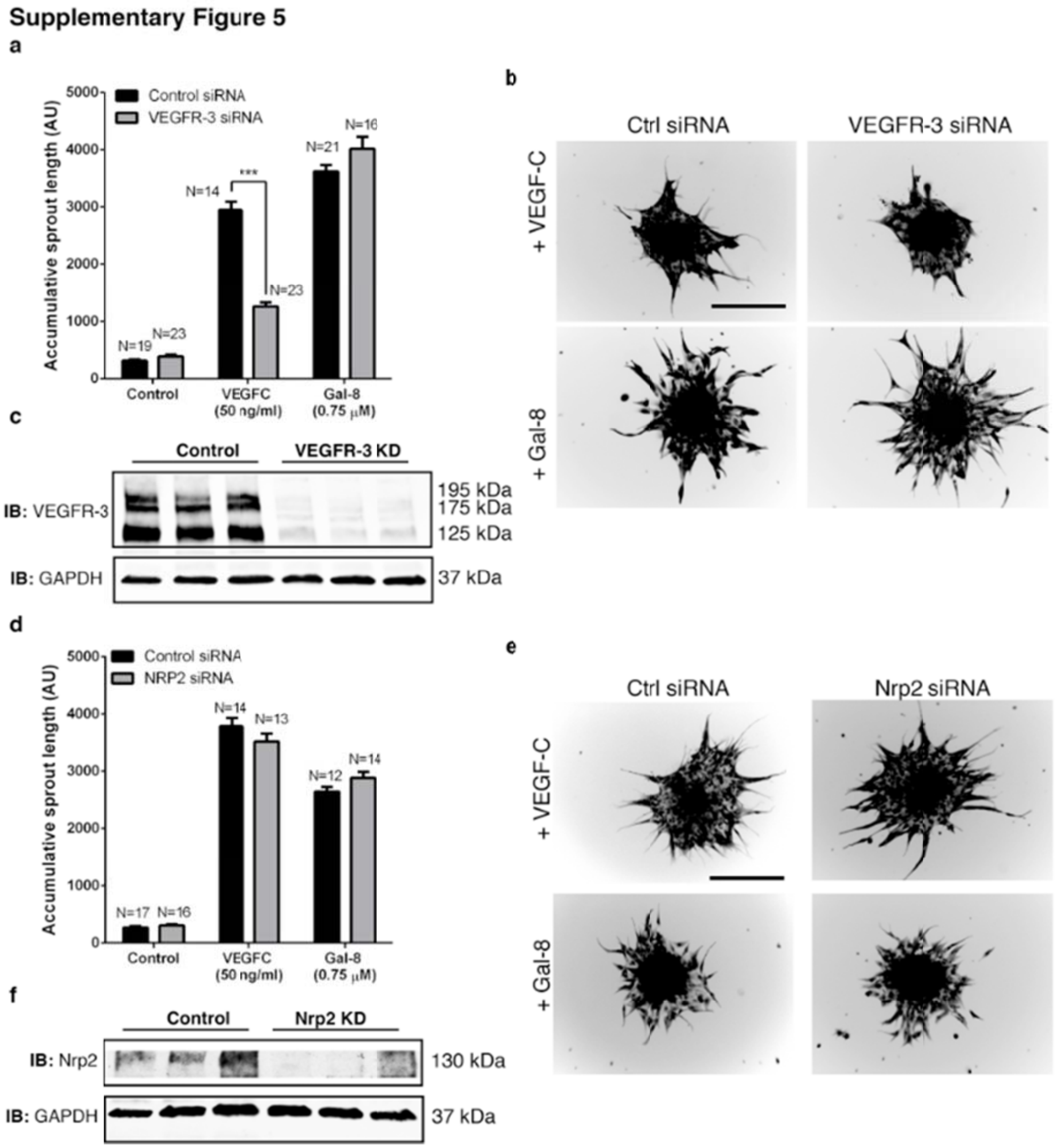

Supplementary figure 5. VEGFR-3 and Neuropilin-2 (Nrp2) knockdown has little effect on galectin-8-induced lymphangiogenesis. (**a,b,d,e**) LEC spheroids prepared using primary LECs transfected with control, VEGFR-3 siRNA (a,b) or Nrp2 siRNA(d,e) were treated with galectin-8 (0.75 µM) or VEGF-C (50 ng per ml). After 24 hr, accumulated sprout lengths were quantified (a,d). Representative images are shown in the right panel (b,e). Data are plotted as mean ± SEM and analyzed using Student's t test. (**c,f**) Assessment of VEGFR-3 and Nrp2 knockdown efficiency. Cell lysates from control, VEGFR-3 knockdown, or Nrp2 knockdown cells were subjected to SDS-PAGE followed by immunoblotting with anti-VEGFR-3, anti-Nrp2 and anti-GAPDH. Three independent samples were used in the control and VEGFR-3 or Nrp2 knockdown. Bar: 100 µm (b,e).

Supplement Figure 6

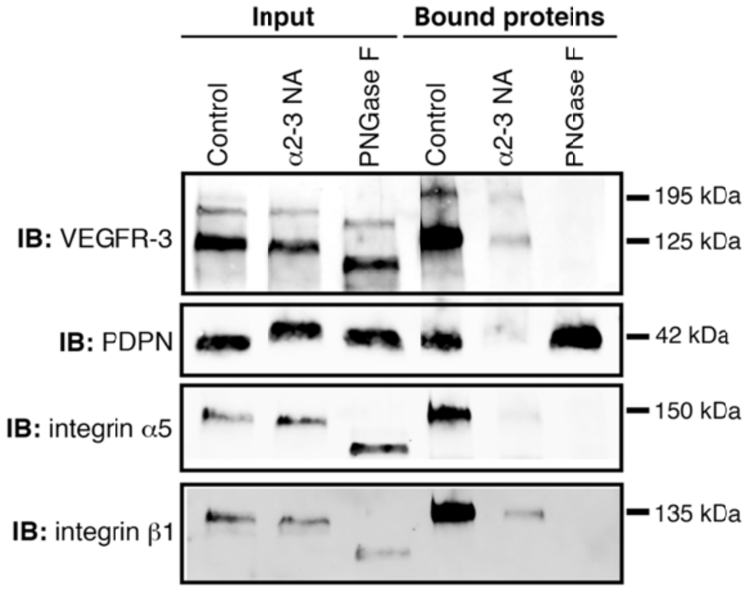

Supplementary figure 6. Galectin-8 interacts with the α2,3-sialyl glycans of PDPN. Primary LECs were incubated with buffer only (control), α2-3 neuraminidase (α2-3NA) that removes α2,3-sialyl glycans, or peptide-N-glycosidase F (PNGase F) that removes complex glycans from N-linked glycoproteins at 37^{0}C for 1 hr. After the incubation, reaction was stopped by adding 400 µl Triton lysis buffer and the reaction mixture was incubated with galectin-8 agarose beads at 4^{0}C overnight. Unbound proteins were removed and the bound proteins were eluted with 20 µl of 2× Laemmli sample buffer and examined along with input by Western blotting. Note that without complex N-glycan, VEGFR-3 and integrins do not interact with galectin-8; PDPN without α2,3- sialyl glycans does not interact with galectin-8. The results are representative of three independent experiments.

Supplementary Figure 7

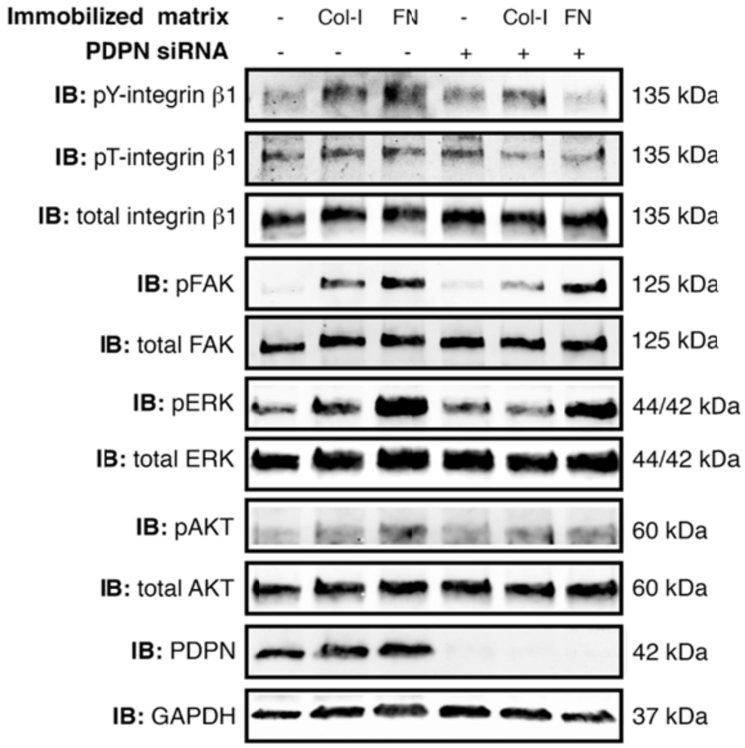

Supplementary figure 7. PDPN modulates integrin-mediated signaling. LECs were transfected with control or PDPN pooled siRNA. The cells were detached 48 hr post-transfection and stimulated with immobilized collagen I (4 µg per cm^2) or fibronectin (2 µg per cm^2), or left in suspension for 15 min at 37°C. Cell lysates were subjected to Western blotting with antibodies indicated in methods.

Supplementary Figure 8

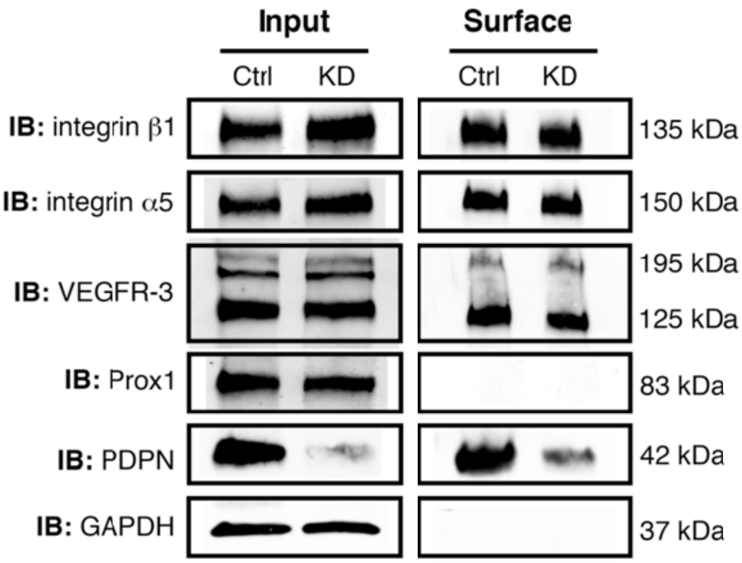

Supplementary figure 8. PDPN knockdown does not alter expression of Prox1 and cell surface expression of integrins and VEGFR-3. Primary LECs were transfected with control siRNA or PDPN pooled siRNA**.** After 48 hr transfection, the cells were incubated with sulfo-NHS-Biotin (0.5 mg per ml) to label cell surface proteins. After biotinylation, cells were lysed and aliquots of cell lysates (500 µg) were incubated with streptavidin-conjugated agarose beads at 4^0C, overnight. Unbound proteins were removed and the bound proteins were eluted with 20 µl of 2× Laemmli sample buffer and examined along with whole cell lysate (25 µg) by Western blotting. Representative blots from two independent experiments are shown. Cell surface protein expression of integrins β1, α5, and VEGFR-3 is similar in control and PDPN knockdown cells. Total protein expression of integrins β1 and α5 is increased by ~50%, whereas total protein expression of VEGFR-3 and Prox1 is unaltered.

Supplementary Figure 9

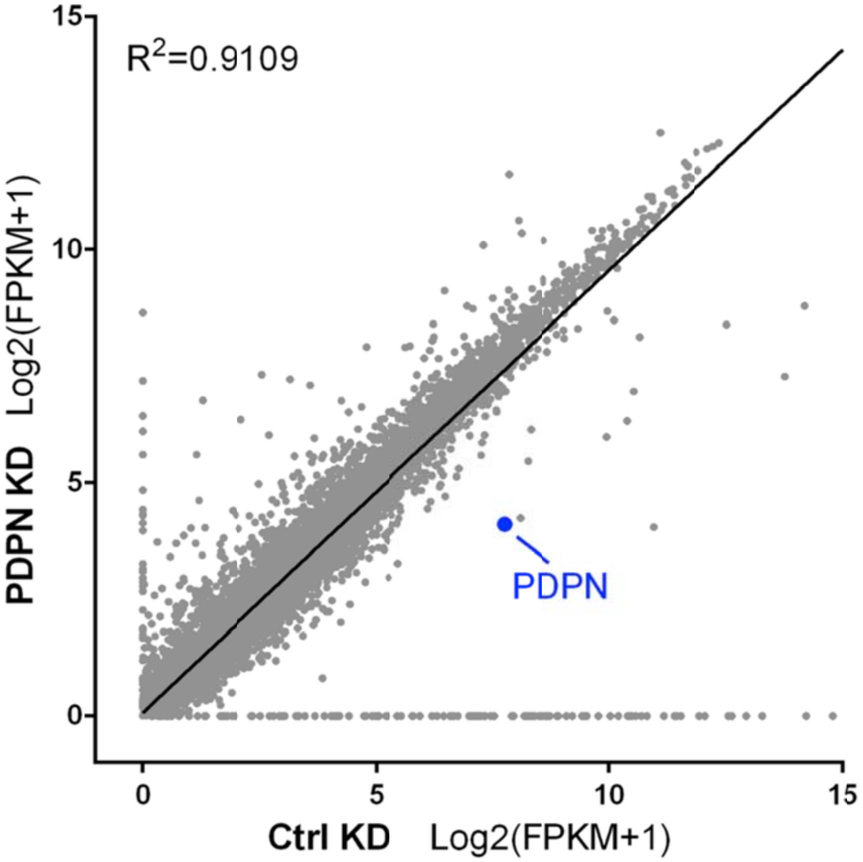

Supplementary figure 9. Gene expression has no significant difference between control and PDPN knockdown LECs. Primary LECs were transfected with PDPN siRNA or AllStars negative control siRNA as described in the main text (*N*=2 for each group). After 48 hr post-transfection, total RNA was extracted and processed for RNA sequencing. Expression values of all genes in both groups were first normalized to FPKM (Fragments Per kilobase of transcript per Million mapped reads) with Cuffdiff software, and transformed to log2(FPKM+1) for generation of the scatterplot with a correlation coefficient. This scatterplot contains all the expression data of annotated genes found in UCSC human reference genome version hg19 (~23,000 genes), with the log transformed values of control group on the X-axis, and PDPN knockdown cells on the Y-axis. (See also Supplementary Data 1). A linear curve fitting was performed and the R^2>0.9, indicating that most genes are not differentially expressed between control and PDPN knockdown LECs. As expected, PDPN is enriched in control cells (FPKM value: 215), whereas it is markedly reduced in PDPN knockdown cells (FPKM value: 16).

Supplementary Figure 10

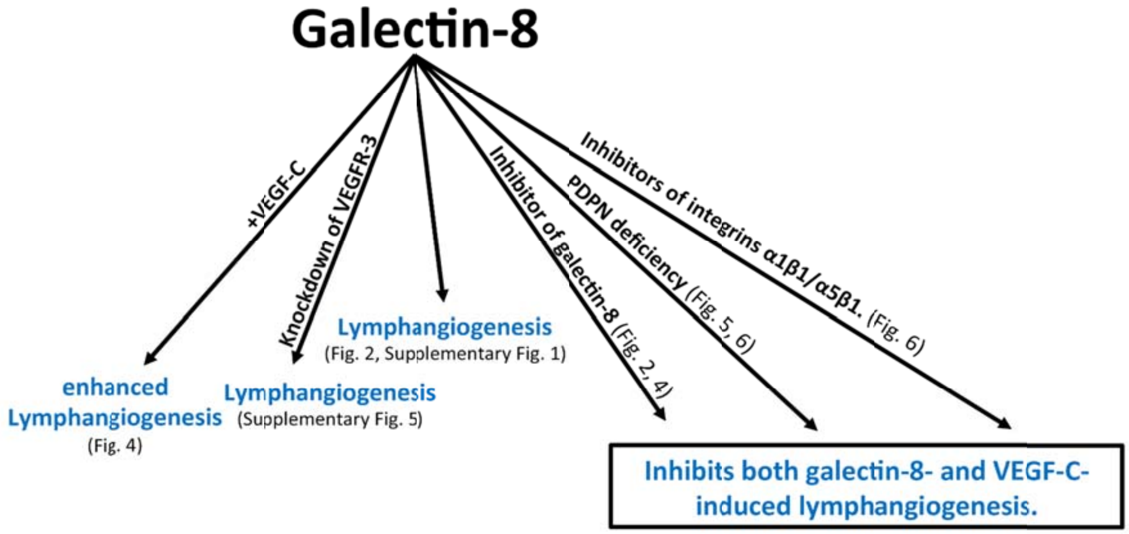

Supplementary figure 10. Schematic representation of mechanistic aspects of galectin-8-induced lymphangiogenesis.

Supplementary Figure 11.

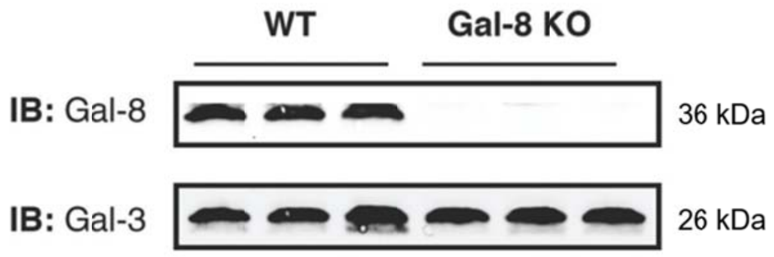

Supplementary figure 11. Confirmation of galectin-8 null status of the knockout mice. Equal amounts of liver tissue lysates (500 µg protein) from the WT and galectin-8 KO mice were incubated with lactosyl Sepharose beads at 4^0C overnight. Unbound proteins were removed by washing the beads with PBS, the bound proteins were eluted by boiling the beads in 20 µL of 2× Laemmli sample buffer and subjected to Western blotting using anti-galectin-8 and anti-galectin-3 on the same blot.

Supplementary Methods

Matrigel plug assay. The matrigel plug assay was used to determine the effect of galectin-8 on lymphangiogenesis in vivo. In this assay, Matrigel (Geltrex^{TM} reduced growth factor basement membrane matrix, Gibco; 300 µl) was mixed with VEGF-C or galectin-8 in 100 µl PBS (final concentration: VEGF-C, 50 ng per ml; galectin-8, 0.5 µM), and was subcutaneously injected into mice (four Matrigel plugs/group). On day 7 post-injection, the Matrigel plugs were harvested and embedded in OCT compound for cryosectioning. The frozen sections (20 µm-thick) were subjected to immunofluorescence staining as described in the Methods section.

Whole-mount analysis. To quantitate the extent of LEC proliferation in *Prox1*-EGFP reporter mice, sustained-release polymer pellets containing galectin-8 (200 ng per pellet) or VEGF-C (100 ng per pellet) were implanted in the corneas of *Prox1*-GFP reporter mice. On day 7 post-surgery, corneas were harvested and fixed in 4% paraformaldehyde/PBS, 30 min, 4°C. The corneas were stained with eFluor 570-anti-mouse Ki67 (clone SolA15, eBioScience, 1:100) in 5% rat serum/5% donkey serum/0.3% Triton X-100/PBS overnight, 4°C. After several washes with 0.3% Triton X-100/PBS, the corneas were flattened and mounted with VECTASHIELD mounting medium (Vector Laboratories) and evaluated by Leica TCS SPE confocal imaging system (Leica). Proliferative LECs (*Prox1*-$GFP^{+}Ki67^{+}$, yellow) and quiescent LECs (*Prox1*-$GFP^{+}Ki67^{-}$, green) in the lymphangiogenic area were enumerated and expressed as percentages.

In vitro proliferation assay. Primary LECs (5,000 cells) were seeded onto gelatin-coated 96-well plates overnight. Quintuplicate wells were treated with VEGF-C (50 ng per ml) or varying concentrations of galectin-8 in 1% FBS/EBM-2 medium. After 48 hr, cell proliferation was

determined by the WST-1 cell proliferation assay kit (Cayman Chemical) according to manufacturer's instructions. Absorbance of each sample was measured using the FilterMax F5 microplate reader (Molecular Devices) at a wavelength of 450 nm. Results were normalized to control wells incubated in media alone to generate percent-change in proliferation activity.

LEC tube formation assay. Primary LECs (3 x 10^5 cells in 200 µl serum-free EBM-2 medium/well) labeled with Calcein AM (2 µg per ml) were seeded into a 48-well plate precoated with 100 µl Matrigel. After allowing the cells to adhere to the Matrigel for 30 min, duplicate wells were treated with VEGF-C or varying concentrations of galectin-8 in 200 µl serum-free EBM-2. After 6 hr incubation, the capillary tube structures were imaged under EVOS FL fluorescence microscope. Tube length was quantified in ImageJ by drawing a line along each tube and the lengths of the lines in pixels were combined for each field. Results were normalized to control cells incubated in media alone to generate percent-change in tube formation activity. Data from 3 independent experiments are shown.

Preparation of HSV-1. The HSV-1 McKrae strain, a stromal disease-causing, neurovirulent HSV-1 strain was used for challenging the corneas. HSV-1 was propagated in Vero cells as described earlier[2]. Briefly, Vero cells were growth to confluence in T150-cm^3 culture flask and infected with 2x10^6 pfu of virus stock in 1.5mL. The flak was tilt every 10 min for 1hr. Then, growth media (MEM plus 5% FBS) was added and cultured for 3 days at 37°C. Afterward, the maximum viral cytopathic effect is expected and the infected cells are extracted after 2-3 cycle of cell lysis by using the GentleMAC dissociator (Miltenyi Biotec Inc, San Diego, CA). The lysate was clarified by centrifuging for 10 min at 3,500 rpm at 4°C, followed by a spin down at

17,000 rpm for 30 min at 4°C. The virus pellet was re-suspend, aliquoted and stored at -80°C. Virus titer was determined by standard plaque assay after the infection of Vero cells.

Glycosidase treatment. To determine whether treatment with neuraminidase inhibits VEGFR-3 and PDPN interaction with galectin-8, primary LECs (3×10^5 cells) were lifted with StemPro® Accutase® cell dissociation reagent, resuspended in 100 µl of PBS with 200 units of α2-3 neuraminidase (BioLabs), or 100 µL of G7 reaction buffer containing 2,000 units of peptide-N-glycosidase F (BioLabs) and 3 U of DNase I (Fisher). The reaction mixtures were incubated at 37^0C for 1 hr. Cell lysates were then subjected to affinity precipitation using galectin-8-conjugated agarose beads and bound proteins were analyzed by Western blot analysis using anti-VEGFR-3, anti-PDPN, anti-integrin α5, and anti-integrin β1 as described in the Methods section.

Analysis of cell surface expression of VEGFR-3, PDPN and integrins by the surface protein biotinylation method. Sulfo-NHS-Biotin (Thermo Scientific) does not permeate cell membranes and is used to label cell surface proteins. LECs transfected with control siRNA or PDPN siRNA were incubated with sulfo-NHS-Biotin (0.5 mg per ml) in PBS at 4°C for 1 hr. At the end of the incubation, cells were washed with iced PBS and excess biotin reagent and byproducts were quenched by 5 min incubation with 50 mM Tris-HCl in PBS (pH 7.4). Then, the cells were lysed with RIPA buffer supplemented with a protease inhibitor cocktail, and cell lysates (500 µg) were incubated with 35 µl of Streptavidin-conjugated agarose beads (Invitrogen) at 4°C, overnight. The bound proteins along with whole cell lysates (25 µg) were analyzed by Western blot analysis using anti-VEGFR-3, anti-PDPN, anti-integrin α5, anti-integrin β1, anti-Prox1 (AF2727, 1 µg per ml, R&D), and anti-GAPDH as described in the Methods section.

Transcriptome analysis using RNA sequencing (RNA-Seq). RNA-Seq based gene expression analysis were used to investigate the effect of PDPN knockdown on the global physiological status of LECs. By comparing the gene expression results obtained between control and PDPN knockdown groups, the genes that are differentially expressed can readily be identified. Primary LECs were transfected with AllStar Negative siRNA and PDPN siRNA as described in the main text (N=2 for each group). After 48 hr, total RNA was isolated using RNeasy Mini Kit (Qiagen) and the resulted RNA samples were treated with DNase I to eliminate DNA contamination according to the manufacturer's instructions. The quantity and integrity of the RNA was assessed by Agilent BioAnalyzer 2100 with RNA Pico Assay. Purified RNA were sent to Tufts University Genomics Core for library preparation, library sequencing and data analysis. For the construction of RNA-Seq library, a minimum of 50ng high quality RNA (RNA integration number of 8 or above, as estimated by the BioAnalyzer) was used as input material. Briefly, RNA samples were amplified and converted to double stranded cDNA using NuGEN Ovation RNA-Seq system V2. The cDNA samples were sonicated to an average fragment size of 200 – 400 bp with a Covaris M220 sonicator. The sonicated DNA fragment will be used as input for library preparation using Illumina TruSeq Nano DNA Sample pre kit per manufacturer's instructions. The quality of the resulted library will be checked on a BioAnalyzer 2100. Libraries that pass quality check process were sequenced on an Illumina HiSeq 2500 with single read 100 bases format using the V4 High Output chemistry. Four libraries were multiplexed and sequenced on each lane, with a read yield of 20 – 30 million reads per sample. The results were acquired as demultiplexed and compressed fastq files. The quality of the raw sequencing output was confirmed with FastQC analysis. The resulted read was then mapped against annotated UCSC human reference genome version hg19

(~23,000 genes) using RNA-Seq aligner such as Tophat or STAR aligner[3]. The resulted files were used as differential expression analysis using Cufflink or DESeq pipeline[4]. Global gene expression data was shown in Supplementary Data 1.

Supplementary References

1 Murakami, M. *et al.* VEGFR1 tyrosine kinase signaling promotes lymphangiogenesis as well as angiogenesis indirectly via macrophage recruitment. *Arterioscler Thromb Vasc Biol* **28**, 658-664, doi:10.1161/ATVBAHA.107.150433 (2008).

2 Blaho, J. A., Morton, E. R. & Yedowitz, J. C. Herpes simplex virus: propagation, quantification, and storage. *Curr Protoc Microbiol* **Chapter 14**, Unit 14E 11, doi:10.1002/9780471729259.mc14e01s00 (2005).

3 Dobin, A. *et al.* STAR: ultrafast universal RNA-seq aligner. *Bioinformatics* **29**, 15-21, doi:10.1093/bioinformatics/bts635 (2013).

4 Trapnell, C. *et al.* Transcript assembly and quantification by RNA-Seq reveals unannotated transcripts and isoform switching during cell differentiation. *Nat Biotechnol* **28**, 511-515, doi:10.1038/nbt.1621 (2010).
